# Dye–catalyst dyads for photoelectrochemical water oxidation based on metal-free sensitizers†

**DOI:** 10.1039/d0ra10971a

**Published:** 2021-01-28

**Authors:** Cristina Decavoli, Chiara L. Boldrini, Vanira Trifiletti, Sally Luong, Oliver Fenwick, Norberto Manfredi, Alessandro Abbotto

**Affiliations:** Department of Materials Science, INSTM Unit, Solar Energy Research Center MIB-SOLAR, University of Milano – Bicocca Via R. Cozzi 55 I-20125 Milano Italy alessandro.abbotto@unimib.it norberto.manfredi@unimib.it; School of Engineering and Materials Science (SEMS), Queen Mary University of London Mile End Road London E1 4NS UK

## Abstract

Dye-Sensitized Photoelectrochemical Cells (DS-PECs) have been emerging as promising devices for efficient solar-induced water splitting. In DS-PECs, dyes and catalysts for water oxidation and/or reduction are typically two separate components, thus limiting charge transfer efficiency. A small number of organometallic dyes have been integrated with a catalyst to form an integrated dye–catalyst dyad for photoanodes, but until now no dyads based on metal-free organic dyes have been reported for photoanodes. We herein report the first example of dyad-sensitized photoanodes in DS-PEC water splitting based on metal-free organic dyes and a Ru catalyst. The di-branched donor–π–acceptor dyes carry a donor carbazole moiety which has been functionalized with two different terminal pyridyl ligands in order to coordinate a benchmark Ru complex as a water oxidation catalyst, affording water oxidation dyads. The two dyads have been fully characterized in their optical and electrochemical properties, and XPS has been used to confirm the presence of the catalyst bonded to the dye anchored to the semiconductor anode. The two dyads have been investigated in DS-PEC, showing an excellent faradaic efficiency (88% average across all cells, with a best cell efficiency of 95%), thus triggering new perspectives for the design of efficient molecular dyads based on metal-free dyes for DS-PEC water splitting.

## Abstract 2

Dye–catalyst dyads based on metal-free dyes were prepared for dye-sensitized photoanodes in photoelectrochemical water splitting, showing a top ranked faradaic efficiency for O_2_ generation up to 95%.

## Graphical abstract

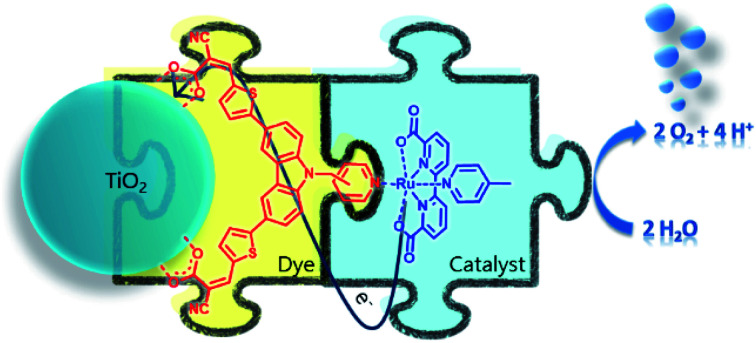

## Introduction

In the search for an alternative to fossil fuels, hydrogen is gaining increasing interest in the scientific community and the automotive-aerospace sector, because its combustion generates only water with a zero-carbon footprint.^1–3^ Hydrogen is today mainly produced from methane *via* the steam-reforming reaction.^4^ It is, therefore, critical to develop sustainable and clean processes to produce hydrogen. In this scenario, solar-driven water splitting has been playing an increasing role, with particular reference to the direct conversion from water and sunlight *via* photocatalysis (PC) or photoelectrochemical cells (PEC).^5–7^ A PEC is a device composed of two electrodes soaked in a water-based electrolyte, where at least one of them is photoactivated. The photoelectrodes typically consist of a wide bandgap semiconductor (SC), which can be sensitized adequately by a dye to extend the absorbed spectrum into the visible region.^8,9^ In the case of a DS-PEC with only a photoactive anode, a platinum wire is used as a passive cathode. The sensitizer is adsorbed or chemically bonded onto the SC surface, while the WOC, typically a ruthenium complex, could be either dispersed in aqueous media^10,11^ or adsorbed onto the SCs surface.^12,13^ Such arrangements are not optimal for the whole process. In the former case, the device efficiency strongly depends on the diffusion of the catalyst near the electrode. In contrast, in the latter one, the adsorption of the catalyst on the SC surface may compete with the absorption of the dye, limiting the light-harvesting capability. An innovative way out is to design an integrated dye–catalyst supramolecular system, also referred to as a dyad, where the molecular sensitizer and the catalyst are bound together as a single molecular unit. In this way, the drawbacks of the two previous arrangements are circumvented, providing more efficient light-harvesting and a faster charge transfer between the two components. Despite its great potential, only a few examples of molecular dyads for photoanodes have been so far reported in the literature. These examples are limited to organometallic compounds in the dye part of the dyad (such as Ru complexes or porphyrins).^14–16^ The absence in the literature of organic dyes in dye–WOC dyads for photoanodes is surprising, considering the emerging role of metal-free dyes in the fields of solar energy conversion.^8,17–19^ Only two examples of dyads containing organic dyes have been so far reported, but both referred to photoactive cathodes.^20,21^

**Fig. 1:**
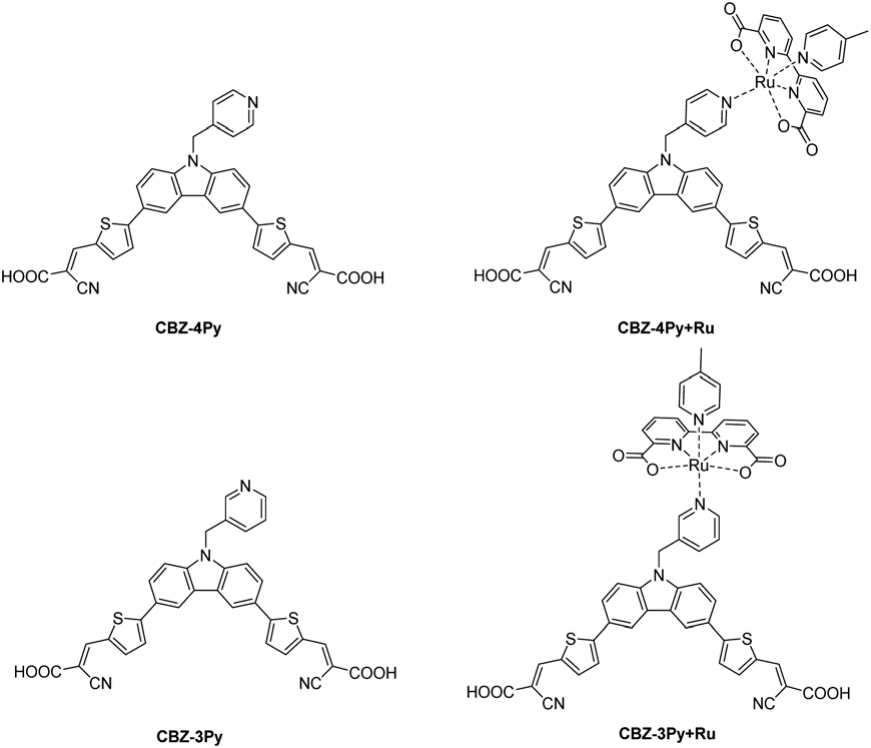
Structures of the investigated dyes and corresponding dye–catalyst dyads.

We thus decided to investigate a dyad where a metal-free sensitizer is covalently bonded to a typical WOC, often used as a catalyst benchmark (a derivative of [Ru(bda)(pic)_2_] (bda = 2,2′-bipyridine-6,6′-dicarboxylate; pic = 4-picoline)).^22^ As a metal-free sensitizer, we have selected a di-branched donor–acceptor D–(π–A)_2_ molecule that we have previously investigated, as a class, in the field.^10,23,24^ The donor moiety was functionalized with a pyridine ligand, which is then used to coordinate the Ru centre of the WOC complex. To study the effect of different spatial arrangements between the dye and the catalyst, two pyridine ligands, with a *para* and *meta* relative position between the coordinating nitrogen atom and a methylene linker to the dye, were envisaged, affording the CBZ-4Py and CBZ-3Py precursors, respectively (Fig. 1). The methylene linker of the picolyl functionality was introduced to break the π-conjugation path between the dye and the catalyst. This ensures that the dyad is constituted by two separated π-conjugated entities, thus avoiding perturbation of the optical, electrochemical, and catalytic properties of the two components, and circumventing charge recombination processes.^16,25^ The two corresponding molecular dyads CBZ-4Py + Ru and CBZ-3Py + Ru were obtained through a two-step procedure directly on the electrode and fully characterized in their optical and electrochemical properties.

When used in water splitting PEC, the investigated dyads showed high faradaic efficiencies (FE), with the *meta*-pyridine dyad CBZ-3Py + Ru showing the highest FE.

As the metal-free D–(π–A)_2_ component of the dyads, we selected a carbazole (CBZ) derivative, which has been functionalized with pyridine linkers. The CBZ donor building block has been selected as a versatile sulphur-free alternative to the commonly used phenothiazine scaffold.^8,18,26^ The central pyrrole-like ring ensures the strong electron-donor character.^27^ The presence of the two benzofused benzene rings can be exploited to easily build the two (π–A) arms carrying the terminal cyanoacrylic acceptor-anchoring groups. Moreover, the NH group of CBZ can be easily functionalised with the pyridine linker needed to couple to the WOC. CBZ-based sensitizes have been successfully employed in PC and PEC water oxidation and hydrogen generation.^10,23^ As WOC, we selected a close derivative of the ruthenium-based benchmark for DS-PEC, [Ru(bda)(pic)_2_], which performed well in many literature examples.^28^

## Experimental

### General information

#### Spectroscopic and electrochemical investigation of dyes

Absorption spectra were recorded with a V-570 Jasco spectrophotometer. UV-O_3_ treatment was performed using Novascan PSD Pro Series – Digital UV Ozone System. The thickness of the layers was measured utilizing a VEECO Dektak 8 Stylus Profiler. Cyclic Voltammetry (CV) was carried out at a scan rate of 50 mV s^−1^, using a Bio-logic SP-240 potentiostat in a three-electrode electrochemical cell under Ar. The working, counter, and the pseudo-reference electrodes were an FTO glass for the dyes in solution or a sensitized 3 μm-thick TiO_2_ film, a Pt wire and an Ag/Ag^+^ electrode (0.01 M AgNO_3_, 0.1 M TBAClO_4_ in ACN) or an Ag/AgCl electrode (3 M KCl), respectively. The preparation and sensitization of the 3.5 μm thick TiO_2_ film are described below. The Pt wire was sonicated for 15 min in deionized water, washed with 2-propanol, and cycled for 50 times in 0.5 M H_2_SO_4_ before use. The Ag/Ag^+^ and the Ag/AgCl pseudo-reference electrodes were calibrated by adding ferrocene (10^−3^ M) to the test solution after each measurement (potentials measured *versus* Fc/Fc^+^ and converted to NHE by addition of +0.63 V;^29^ NHE converted to vacuum by addition of −4.6 V).^30^

#### Preparation of photoanodes

The photoanodes have been prepared as described below, adapting a procedure reported in the literature.^31^ All the containers used were in glass or Teflon and were treated with EtOH and 10% HCl before use to exclude metal contamination. FTO glass was cleaned in a detergent solution for 15 min using an ultrasonic bath, rinsed with pure water and EtOH. After treatment in a UV-O_3_ system for 18 min, a transparent active layer of 0.8 cm^2^ was screen-printed using Dyesol 18NR-T active transparent TiO_2_ paste. The coated films were thermally treated at 125 °C for 6 min, 325 °C for 10 min, 450 °C for 15 min, and 500 °C for 15 min. The heating ramp rate was 5–10 °C min^−1^.

FTO plates coated with 3.5 μm transparent TiO_2_ film, prepared as described above, were treated in a UV-O_3_ system for 20 min at room temperature, then immersed into a 2 × 10^−4^ M solution in EtOH of the dye + catalyst precursor for 3 h at room temperature in the dark. The stained substrates were rinsed with EtOH and dried with a stream of dry nitrogen. The UV-vis spectra, CV and LSV were recorded in comparison with a bare 3.5 μm transparent TiO_2_ film.

#### X-ray photoelectron spectroscopy (XPS) measurements

XPS was performed on Thermo Scientific K-Alpha X-ray photoelectron spectrometer with a monochromatic Al Kα X-ray source under high vacuum (2 × 10^−8^ mbar), using an electron flood gun to avoid sample charging. The data fitting was performed by Thermo Avantage v5.9911 software, using mixed Gauss–Lorentz functions.

#### IPCE, LHE and APCE measurements

IPCE measurements were performed using a monochromator (JASCO) illuminating on the active area of the working electrode (0.80 cm^2^). The light intensity was monitored using a reference Si cell photodiode (THORLABS, S120VC) and corrected to calculate the IPCE values. The photocurrent was measured using an AUTOLAB PGSTAT302N potentiostat in a three-electrode purposely designed photoelectrochemical cell and the data collected with GPES electrochemical interface (EcoChemie). LHE spectra have been recoded, using the same samples, with a spectrophotometer Jasco V-570. APCE were calculate according to the relationship:IPCE(*λ*) = LHE(*λ*) × APCE(*λ*)

#### Oxygen evolution quantification by collector–generator technique

The dyad-sensitized photoanode was illuminated under an applied bias, thus acting as an O_2_ generator. An FTO electrode (previously cleaned *via* 15 min sonication in EtOH) was sandwiched to the photoanode (with both conducting sides facing inward), in order to be used as the collector, *i.e.* the electrode at which the reduction of the evolved O_2_ takes place. The sandwiched device was held together by 4 layers of unstretched parafilm (*ca.* 400 μm spacing), sealed by pressing at 65 °C for 60 s. The two lateral sides of the C-G sandwich were left open, allowing for the filling of void space between electrodes with electrolyte solution by capillary forces. Both the photoanode generator and the FTO collector were contacted using Cu tape (also covered by the parafilm layers), and respectively connected to the two working electrodes of a bipotentiostat (Autolab PGSTAT302N). An Ag/AgCl electrode and a Pt wire were used as the reference and the counter electrode. The sandwich was then immersed in degassed Na_2_SO_4_ 0.1 M solution (pH 5.8) and illuminated with a 200 W xenon lamp (using a LOT-Oriel xenon white light source, equipped with a 420 nm cut-off filter to minimize TiO_2_ contribution and an IR filter to avoid cell warming). The generator electrode was held at 0.3 V *vs.* Ag/AgCl bias (∼0.5 V *vs.* NHE), while the collector at −0.8 V *vs.* Ag/AgCl (∼−0.6 V *vs.* NHE), identified in literature as the optimal O_2_ reduction potential.^32^

In a typical experiment, the working electrodes' currents were recorded for 300 s in the dark, then during 300 s of illumination, and in the dark for an additional 300 s. The procedure allows the diffusion of oxidation products across the solution. The faradaic efficiency for O_2_ production, *η*_O_2__, can be calculated by:
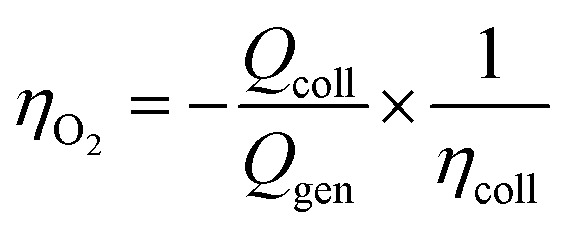
where *Q*_coll_ is the integrated current measured at the collector electrode, *Q*_gen_ is the integrated photocurrent measured at the generator electrode, and *η*_coll_ is the collector efficiency. The latter must be quantified under the specific set-up and experimental conditions used; in our case 82%, in good agreement with the one reported by the Finke^33^ group on a very similar set-up (*i.e.* with a small gap between the generator and collector).

### Synthesis

NMR spectra were recorded with a Bruker AMX-500 spectrometer operating at 500.13 MHz (^1^H) or Bruker Avance Neo spectrometer operating at 400 MHz (^1^H) and 100 MHz (^13^C). Coupling constants are given in Hz. ATR FT-IR spectra were recorded with a PerkinElmer Spectrum 100. High resolution mass spectra have been recorded with an Agilent 6230B Time of Flight (TOF) equipped with an electrospray (dual ESI) source. Melting point were recorded with a Büchi Melting Point M-565. Flash chromatography was performed with Merck grade 9385 silica gel 230–400 mesh (60 Å). Reactions performed under inert atmosphere were performed in oven-dried glassware, and a nitrogen atmosphere was generated with Schlenk technique. The conversion was monitored by thin-layer chromatography by using UV light (254 and 365 nm) as a visualizing agent. All reagents were obtained from commercial suppliers at the highest purity grade and used without further purification. Anhydrous solvents were purchased from Acros Organics and used without further purification. Extracts were dried with Na_2_SO_4_ and filtered before removal of the solvent by evaporation. Compounds 9*H*-3,6-dibromocarbazole (1),^34^ 4,4,5,5-tetramethyl-2-[5-(4,4,5,5-tetramethyl-1,3-dioxolan-2-yl)thiophen-2-yl]-1,3,2-dioxaborolane,^35^ [Ru(bda)(DMSO)(MeCN)(pic)]^36^ (bda = 2,2′-bipyridine-6,6′-dicarboxylate, pic = 4-picoline) were prepared according to literature.

#### 3,6-Bis(5-(4,4,5,5-tetramethyl-1,3-dioxolan-2-yl)thiophen-2-yl)-9*H*-carbazole (2)

9*H*-3,6-Dibromocarbazole (415 mg, 1.27 mmol) and Pd(dppf)Cl_2_·CH_2_Cl_2_ (106 mg, 0.13 mmol) were dissolved in dimethoxyethane 2.5 mL and stirred for 15 min under nitrogen atmosphere. Then 4,4,5,5-tetramethyl-2-[5-(4,4,5,5-tetramethyl-1,3-dioxolan-2-yl)thiophen-2-yl]-1,3,2-dioxaborolane (950 mg, 2.81 mmol) and methanol (2.5 mL) were added and the solution was stirred for 15 min under nitrogen atmosphere. In the end, K_2_CO_3_ (1.75 g, 12.7 mmol) was added to the solution and the reaction was performed under microwave irradiation (100 °C, 70 W, 90 min) and then quenched by pouring into a saturated solution of NH_4_Cl (40 mL) and CH_2_Cl_2_ (40 mL). Filtration on Celite and extractions with organic solvent allowed to isolate the crude product, then purified through column chromatography on silica gel (*n*-eptane : AcOEt – 2 : 1). The desired product was isolated as a white solid (383 mg, 0.65 mmol) with 51% yield. ^1^H NMR (500 MHz, CDCl_3_): *δ* (ppm) = 8.30 (s, 2H), 8.09 (s, 1H), 7.68 (dd, *J* = 8.4, 1.6 Hz, 2H), 7.41 (d, *J* = 8.4 Hz, 2H), 7.20 (d, *J* = 3.7 Hz, 2H), 7.14 (d, *J* = 3.6 Hz, 2H), 6.22 (s, 2H), 1.39 (s, 12H), 1.34 (s, 12H).

#### 9-(Pyridin-4-ylmethyl)-3,6-bis(5-(4,4,5,5-tetramethyl-1,3-dioxolan-2-yl)thiophen-2-yl)-9*H*-carbazole (3a)

Compound 2 (270 mg, 0.46 mmol) was dissolved in 12 mL of anhydrous THF in a two-necked flask under N_2_ atmosphere, then the solution was cooled to 0 °C using an ice bath, and NaH 60% (36 mg, 0.92 mmol) was added and the solution was stirred at 0 °C for 1 h. Meanwhile in a becher 4-(bromomethyl)pyridine hydrobromide (177 mg, 0.70 mmol) was dissolved in 25 mL of a saturated aqueous solution of K_2_CO_3_ and 25 mL of Et_2_O. The mixture was extracted with Et_2_O; the organic phase was washed with water and dried on MgSO_4_. The solution is reduced to 1.5 mL and then slowly introduced in the reaction flask. The resulting solution was stirred at rt overnight. The following day, the reaction mixture was concentrated in Rotavapor up to 5 mL. Then, 10 mL of Et_2_O and 40 mL of iced water were added. The light yellow precipitated solid (285 mg 0.42 mmol) was filtered and washed with Et_2_O achieving a 91% yield. ^1^H NMR (500 MHz, CDCl_3_): *δ* (ppm) = 8.52 (d, *J* = 5.9 Hz, 2H), 8.36 (s, 2H), 7.69 (dd, *J* = 8.6, 1.6 Hz, 2H), 7.26 (d, *J* = 8.2 Hz, 2H), 7.21 (d, *J* = 3.6 Hz, 2H), 7.15 (d, *J* = 3.6 Hz, 2H), 7.02 (d, *J* = 5.7 Hz, 2H), 6.21 (s, 2H), 5.51 (s, 2H), 1.39 (s, 12H), 1.33 (s, 12H).

#### 9-(Pyridin-3-ylmethyl)-3,6-bis(5-(4,4,5,5-tetramethyl-1,3-dioxolan-2-yl)thiophen-2-yl)-10*H*-carbazole (3b)

Compound 2 (383 mg, 0.65 mmol) was dissolved in 10 mL of anhydrous THF in a two-necked flask under N_2_ atmosphere, then the solution was cooled to 0 °C using an ice bath and NaH 60% (90 mg, 2.21 mmol) was added. After stirring at 0 °C for 1 h, the 3-(bromomethyl)pyridine hydrobromide (214 mg, 0.85 mmol) was added and the solution was stirred at rt overnight. The following day, the reaction mixture was quenched with iced water, then extracted with CH_2_Cl_2_ and the organic phase was washed with water, dried with Na_2_SO_4_ and concentrated. The desired product was isolated as a light-yellow solid (420 mg 0.61 mmol) with 95% yield. ^1^H NMR (400 MHz, CDCl_3_): *δ* (ppm) = 8.60 (d, *J* = 1.8 Hz, 1H), 8.50 (dd, *J* = 4.8, 1.5 Hz, 1H), 8.32 (d, *J* = 1.4 Hz, 2H), 7.67 (dd, *J* = 8.5, 1.8 Hz, 2H), 7.29 (d, *J* = 8.5 Hz, 3H), 7.19 (d, *J* = 3.6 Hz, 2H), 7.17–7.05 (m, 3H), 6.21 (s, 2H), 5.47 (s, 2H), 1.39 (s, 12H), 1.33 (s, 12H).

#### General procedure A for the cleavage of the protective group for the aldehydic functionality (iii)

Pinacol ester precursor was dissolved in 10% HCl/THF (1 : 2). The mixture was heated at 50 °C for 2 h and then poured into water. K_2_CO_3_ was slowly added until basic pH was reached, and then THF was removed under reduced pressure. The product precipitated was filtered on Hirsh and washed with water.

#### 5,5′-(9-(Pyridin-4-ylmethyl)-9*H*-carbazole-3,6-diyl)bis(thiophene-2-carbaldehyde) (4a)

Product 4a was synthetized according to general procedure A for the cleavage of the protective group using product 3a (140 mg, 0.21 mmol), 15 mL 10% HCl/THF (1 : 2). The desired product was isolated as a yellow solid (91 mg, 0.19 mmol) with 91% yield. ^1^H NMR (400 MHz, DMSO): *δ* (ppm) = 9.92 (s, *J* = 0.9 Hz, 2H), 8.91 (dd, *J* = 1.9, 0.5 Hz, 2H), 8.48 (d, *J* = 6.1 Hz, 2H), 8.09 (d, *J* = 4.0 Hz, 2H), 7.96 (dd, *J* = 8.6, 1.9 Hz, 2H), 7.81 (d, *J* = 4.0 Hz, 2H), 7.74 (d, *J* = 8.6 Hz, 2H), 7.10 (d, *J* = 6.1 Hz, 2H), 5.84 (s, 2H).

#### 5,5′-(9-(Pyridin-3-ylmethyl)-9*H*-carbazole-3,6-diyl)bis(thiophene-2-carbaldehyde) (4b)

Product 4b was synthetized according to general procedure A for the cleavage of the protective group using product 3b (420 mg, 0.61 mmol), 27 mL 10% HCl/THF (1 : 2). The desired product was isolated as a yellow solid (290 mg, 0.60 mmol) with 98% yield. ^1^H NMR (400 MHz, DMSO): *δ* (ppm) = 9.92 (s, 2H), 8.90 (d, *J* = 1.8 Hz, 2H), 8.58 (d, *J* = 1.9 Hz, 1H), 8.46 (dd, *J* = 4.7, 1.5 Hz, 1H), 8.09 (d, *J* = 4.0 Hz, 2H), 7.97 (dd, *J* = 8.6, 1.9 Hz, 2H), 7.85 (d, *J* = 8.7 Hz, 2H), 7.81 (d, *J* = 4.0 Hz, 2H), 7.59–7.48 (m, 1H), 7.30 (dd, *J* = 7.8, 4.8 Hz, 1H), 5.83 (s, 2H).

#### General procedure B for Knoevenagel condensation (iv)

Aldehyde precursor (1 equiv.), cyanoacetic acid (5 equiv.), and piperidine (6 equiv.) were dissolved in dry CHCl_3_ (0.2 M) and warmed to reflux for 8 h. After having the solvent evaporated, a solution of HCl 10% was added, and the mixture was left under magnetic stirring for 5 h at room temperature. The solid that precipitated was filtered and dissolved in a saturated aqueous solution of K_2_CO_3_. The new solid that precipitated was filtered and dissolved in a solution of citric acid 1 M. Then, the new solid that precipitated was filtered and washed with water.

#### 3,3′-(5,5′-(9-(Pyridin-4-ylmethyl)-9*H*-carbazole-3,6-diyl)bis(thiophene-5,2-diyl))bis(2-cyanoacrylic acid) (CBZ-4Py)

CBZ-4Py was synthetized according to general procedure B for Knoevenagel condensation using product 4b (171 mg, 0.35 mmol), cyanoacetic acid (297 mg, 3.5 mmol), piperidine (358 mg, 4.2 mmol) and 10 mL of dry CHCl_3_. The reaction was quenched with 20 mL of 10% HCl solution. A dark red solid (170 mg, 0.28 mmol) was isolated as the product with 80% yield. Due to low solubility, addition of citric acid (CA) was necessary to improve the solubilization of the compound and to record better NMR spectra. Mp = 220 °C (dec) ^1^H NMR (400 MHz, DMSO) *δ* 8.80 (d, *J* = 1.3 Hz, 2H), 8.52–8.41 (m, 4H), 8.01 (d, *J* = 4.0 Hz, 2H), 7.87 (dd, *J* = 8.6, 1.4 Hz, 2H), 7.80 (d, *J* = 3.9 Hz, 2H), 7.70 (d, *J* = 8.7 Hz, 2H), 7.10 (d, *J* = 5.4 Hz, 2H), 5.80 (s, 2H), 2.75 (d, *J* = 15.4 Hz, 4H, CA), 2.65 (d, *J* = 15.4 Hz, 4H, CA). ^13^C NMR (101 MHz, DMSO) *δ* 175.32 (CA), 171.77 (CA), 164.32, 154.79, 150.38, 146.77, 146.64, 141.90, 141.77, 134.24, 125.75, 125.18, 124.77, 123.48, 122.12, 119.70, 117.31, 111.22, 98.34, 72.77 (CA), 45.41, 43.37 (CA). FT-IR *ν*/(cm^−1^): 2921, 2214 (s), 1702 (s), 1573 (s), 1421 (s), 1199 (s), 793 (s). HRMS (dual-ESI) *m*/*z*: calcd for [M − H]^−^ C_34_H_20_N_4_O_4_S_2_: 611.0853, found 611.0845; calcd for [M − H–CO_2_]^−^ 567.0955, found 567.0949; calcd for [M − H–2CO_2_]^−^ 523.1057, found 523.1048.

#### 3,3′-(5,5′-(9-(Pyridin-3-ylmethyl)-9*H*-carbazole-3,6-diyl)bis(thiophene-5,2-diyl))bis(2-cyanoacrylic acid) (CBZ-3Py)

CBZ-3Py was synthetized according to general procedure B for Knoevenagel condensation using product 4d (290 mg, 0.61 mmol), cyanoacetic acid (690 mg, 8.14 mmol), piperidine (831 mg, 9.77 mmol) and 30 mL of dry CHCl_3_. The reaction was quenched with 10 mL of 10% HCl solution. A dark red solid (200 mg, 0.33 mmol) was isolated as the product with 60% yield. Mp = 221 °C (dec) ^1^H NMR (400 MHz, DMSO): *δ* (ppm) = 8.83 (d, *J* = 1.4 Hz, 2H), 8.59 (s, 1H), 8.53–8.40 (m, 3H), 8.04 (d, *J* = 4.1 Hz, 2H), 7.93 (dd, *J* = 8.6, 1.6 Hz, 2H), 7.87–7.82 (m, 4H), 7.54 (d, *J* = 8.0 Hz, 1H), 7.31 (dd, *J* = 7.8, 4.8 Hz, 1H), 5.82 (s, 2H). ^13^C NMR (101 MHz, DMSO) *δ* 164.27, 154.90, 149.29, 148.82, 146.90, 142.05, 141.69, 135.05, 134.22, 133.31, 125.77, 125.12, 124.81, 124.29, 123.51, 119.74, 117.26, 111.38, 98.16, 44.06. FT-IR *ν*/(cm^−1^): 2919, 2213 (s), 1701 (s), 1575 (s), 1423 (s), 1208 (s), 790 (s). HRMS (dual-ESI) *m*/*z*: calcd for [M − H]^−^ C_34_H_20_N_4_O_4_S_2_: 611.0853, found 611.0844; calcd for [M − H–CO_2_]^−^ 567.0955, found 567.0947; calcd for [M − H–2CO_2_]^−^ 523.1057, found 523.1046.

## Results and discussion

### Synthesis

**Scheme 1:**
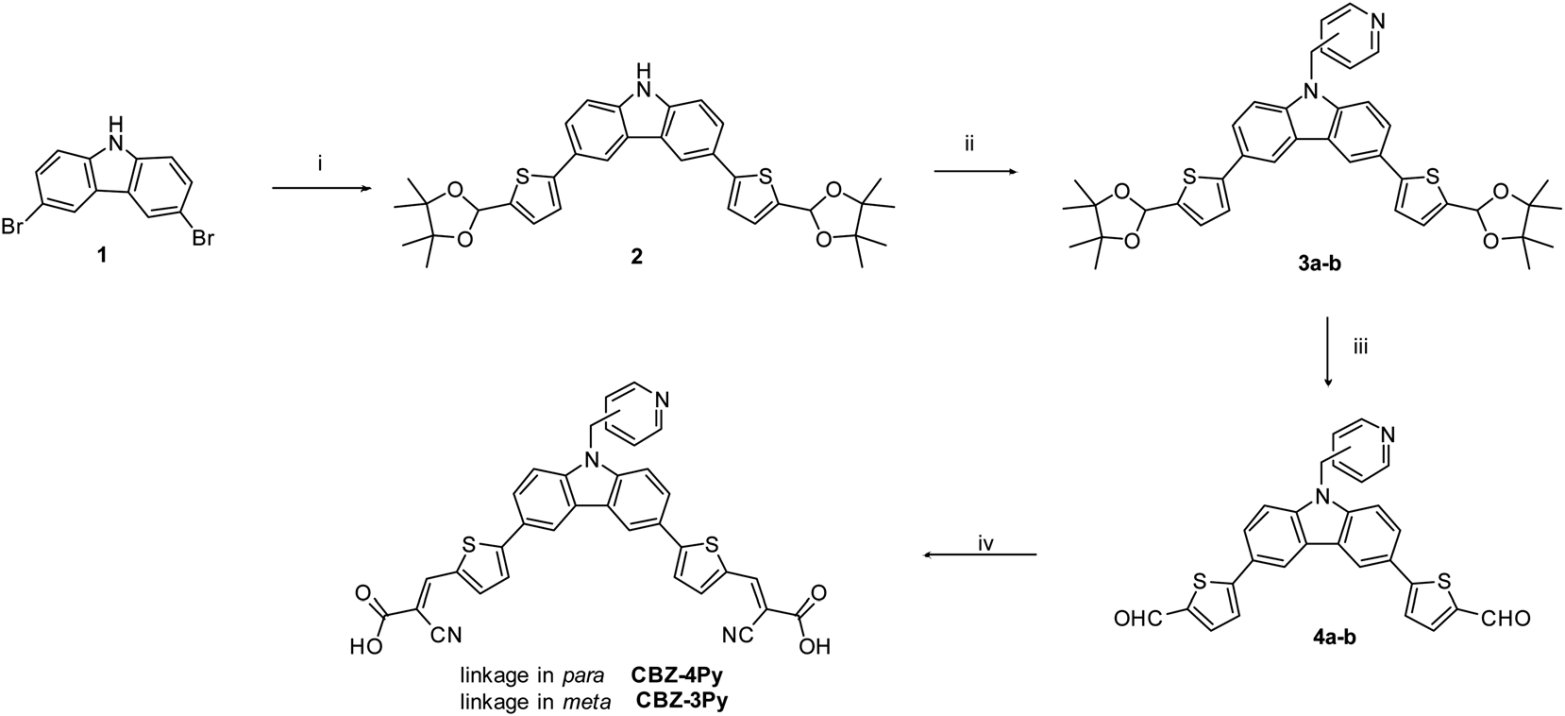
Synthetic pathway for CBZ-4Py and CBZ-3Py. Reagents and conditions: (i) 4,4,5,5-tetramethyl-2-(5-(4,4,5,5-tetramethyl-1,3-dioxolan-2-yl)thiophen-2-yl)-1,3,2-dioxaborolane, Pd(dppf)Cl_2_·CH_2_Cl_2_, K_2_CO_3_, DME : MeOH (1 : 1), microwave 100 °C, 70 W, 60 min; (ii) 4-(bromomethyl)pyridine hydrobromide or 3-(bromomethyl)pyridine hydrobromide, NaH 60%, THF anhydrous, 0 °C, 30 min and then rt, overnight; (iii) HCl 10% : THF (1 : 2), 50 °C, 2 h; (iv) cyanoacetic acid, piperidine, CHCl_3_ dry, reflux, 8 h.

The synthesis of the dyes CBZ-4Py and CBZ-3Py functionalised with the two pyridine linkers is presented in Scheme 1. The Suzuki–Miyaura cross-coupling between commercially available 9*H*-3,6-dibromocarbazole and the protected pinacol ester of 5-formyl-2-thienylboronic acid^35^ affords intermediate 2 with protected formyl groups. The deprotonated derivative is then submitted to alkylation with the proper picolylbromide to give the pyridine derivatives 3a and 3b. Subsequent deprotection of the formyl groups and final Knoevenagel condensation with cyanoacetic acid, in the presence of piperidine, yields the desired sensitizers with the pyridine ligand. The new sensitizers have been fully characterised with ^1^H and ^13^C NMR, as well as melting point, FT-IR, and HRMS to check the required structures and purities (see ESI†).

**Scheme 2:**
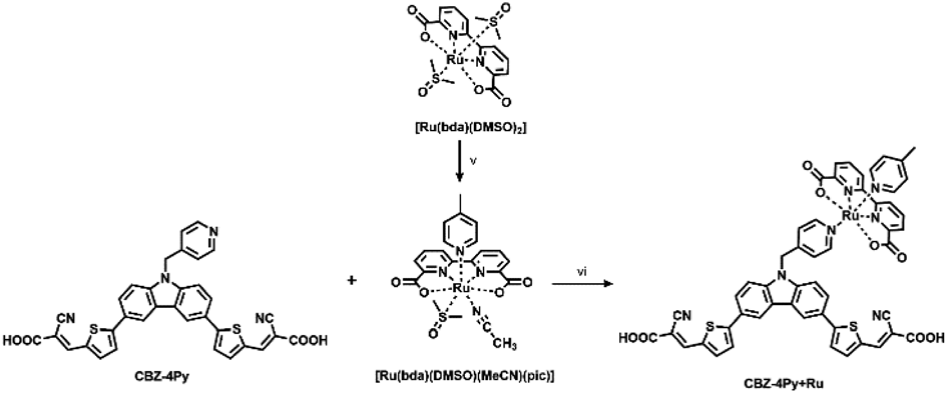
General procedure for the synthesis of the dyads in solution. Reagents and conditions: (v) 4-picoline, MeCN, reflux, 1 h; (vi) MeOH, DMSO, reflux, 1 day.

For the synthesis of the dyads CBZ-4Py + Ru and CBZ-3Py + Ru, we first attempted to apply the synthetic approach used for heteroleptic [Ru(bda)(pic)(ligand)] complexes (Scheme 2).

A stoichiometric amount of the dye was added to [Ru(bda)(DMSO)(MeCN)(pic)],^14,16^ but, unfortunately, the desired product could not be characterized due to good solubility only in coordinating solvents, thus decomposing the Ru complex of the dyad. To overcome this issue, the dyad was completed directly on the electrode surface, starting from the synthetic precursors. A TiO_2_ electrode was soaked into a 2 × 10^−4^ M EtOH/DMSO (9 : 1) dye solution in the presence of a stoichiometric amount of the Ru precursor. To eliminate the unreacted Ru precursor, the photoelectrode was then rinsed with EtOH. Since the Ru precursor does not carry any anchoring group, the final step ensures that the presence of Ru in the rinsed electrode can only be due to the formation of the desired complex with the functionalized dye, in turn, anchored to the SC *via* the acceptor-carboxylic functionality.

### Optical and electrochemical properties

**Fig. 2:**
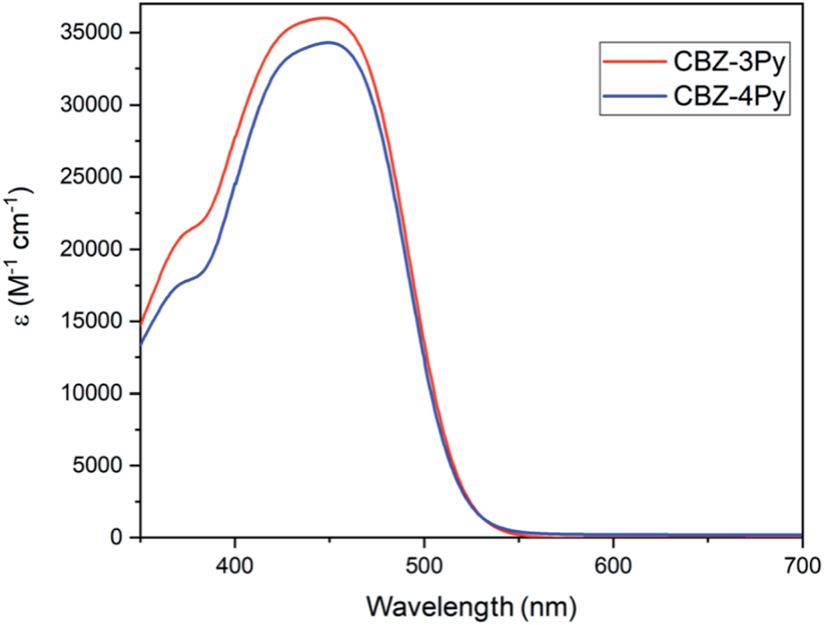
Absorption spectra of dyes in solution.

The absorption spectra (molar absorption coefficient *vs.* wavelength) of the dyes CBZ-4Py and CBZ-3Py in solution (10^−5^ M in DMSO) are shown in Fig. 2 and the main optical parameters are listed in Table 1. As expected, the spectra of the two dyes are almost identical, since the picolyl functionality does not affect the π-framework of the D–(π–A)_2_ dyes. The UV-vis spectra in solution exhibited the main intramolecular charge transfer (ICT) transition at *ca.* 450 nm. The UV-vis analysis of the dyes adsorbed onto a 1 μm transparent TiO_2_ film presented a similar absorption intensity for the ICT band, with the peak at *ca.* 30 nm lower wavelengths. The optical band gaps were estimated using the method of Tauc plot^37^ and listed in Table 1. The normalized spectra of the TiO_2_ films sensitized with the dyads CBZ-4Py + Ru and CBZ-3Py + Ru are shown in Fig. S1 (ESI).† They are in good agreement with the dyes' absorption spectra: the shape in the visible range is dominated by the ICT band of the dye portion. The electrochemical properties of the dyes in solution have been investigated, and the main electrochemical parameters are collected in Table 1.

**Table 1:** Main optical and electronic parameters of dyes in solution and adsorbed onto a TiO_2_ film

Dye	*λ*_max_ a ^,^ b (nm)		*ε* a (M^−1^ cm^−1^)	Dye loading (nmol cm^−2^)	*V*_ox_ a ^,^ b (V *vs.* Fc/Fc^+^) ± 10 mV		HOMO^a^^,^^b^^,^^c^ (eV) ± 0.05 eV		*E* ^opt^_gap_ a ^,^ b (eV)		LUMO^a^^,^^b^^,^^c^ (eV) ± 0.05 eV	
	Soln	Film			Soln	Film	Soln	Film	Soln	Film	Soln	Film
CBZ-4Py	448	417	34 300 ± 100	37.1	0.83	0.59	−6.06	−5.82	2.26	2.12	−3.80	−3.70
CBZ-3Py	447	413	36 000 ± 2000	55.8	0.85	0.78	−6.08	−6.01	2.26	2.14	−3.82	−3.87

a Dye solution 10^−5^ M in DMSO.

b 1 μm transparent TiO_2_ photoanode.

c Fc^+^/Fc = −5.23 eV *vs.* vacuum.

Cyclic voltammetry (CV) profiles (Fig. S2, ESI†) showed irreversible behaviour for the oxidation processes of CBZ-3Py and CBZ-4Py. Differential Pulsed Voltammetry (DPV) (Fig. S3, ESI†) was thus used to determine the oxidation potential from the current peak^38^ and then calculate the HOMO energy levels, which were estimated to be around −6 eV. This value is lower than the electron-donating level of the WOC (−5.8 eV),^39^ and therefore dye regeneration can take place. The LUMO levels have been derived from electrochemical HOMO values and optical bandgaps, calculated as described above. The so calculated LUMO energies are also very similar, and both are at higher energy than that of the CB of TiO_2_ (−4.0 eV *vs.* vacuum), thus ensuring efficient electron injection from the excited dye to the SC. Therefore, the electrochemical study guarantees proper energy levels alignments for electron–hole donation among the dye and other components of the cell.

### Characterisation of the photoanodes

**Fig. 3:**
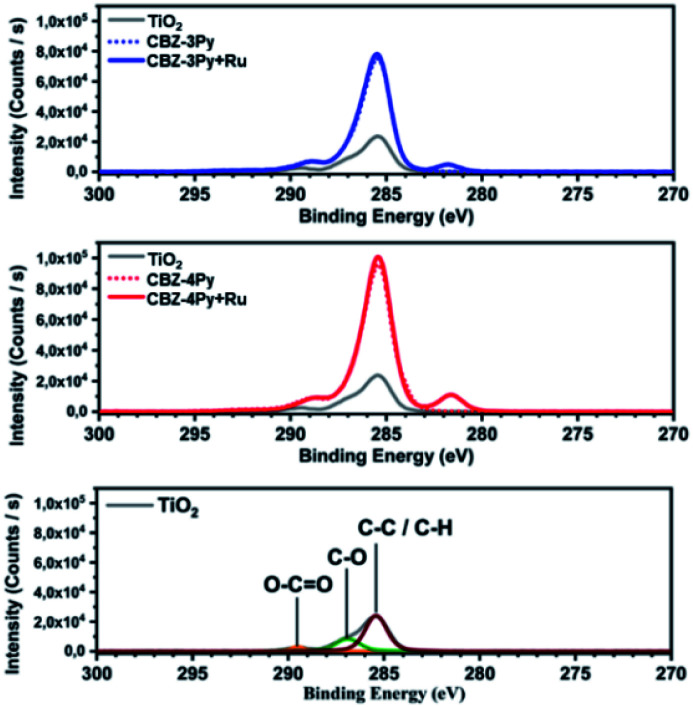
XPS survey spectra of the high-resolution core level for C 1s and Ru 3d region of the investigated dyes and corresponding dyads, compared to the bare TiO_2_.

The electrochemical properties of the dyad-sensitized anodes have been investigated by CV and compared with those of the dye and the reference catalyst [Ru(bda)(pic)_2_] (Fig. S4 and S5, ESI†). The CV profile of the CBZ-4Py + Ru-sensitized electrode presented a redox peak at *E*_1/2_ = +0.70 V *vs.* NHE, which well compares with the reported value for the reference Ru complex (+0.65 V *vs.* NHE).^39^ The CBZ-3Py + Ru-sensitized electrode showed a less intense, but still reversible, redox peak at *E*_1/2_ = +0.77 V *vs.* NHE. It is slightly higher than the reported potential for the redox benchmark, but still comparable to it and other similar systems.^13,25^ In contrast, the dye-sensitized electrode evidenced only the beginning process of the dye oxidation, in agreement with the CV profile of the corresponding *N*-alkylcarbazole derivative^10^ not carrying the terminal pyridine functionality (Fig. S6, ESI†). The electrochemical study of the dyad-sensitized electrodes thus supports the presence of a [Ru(bda)(pic)(ligand)] complex anchored, through the dye, to the SC. X-ray photoelectron spectroscopy (XPS) analysis was performed to confirm the formation of the anchored dyads. The XPS survey spectra (Fig. S7, ESI†) show the presence of sulphur and nitrogen peaks on the TiO_2_ surface, thus supporting the presence of the dye portion. Ruthenium peaks are not clearly resolved in the survey spectra due to the proximity to the carbon peak. The core-electron binding energy of C 1s and Ru 3d_5/2_ are plotted in Fig. 3: C 1s peaks for adventitious carbon are visible at 285.4 eV (C–C/C–H), 286.9 eV (C–O) and 289.5 eV (O–C=O).^40^ After adsorption of the dyads, the C–O peak is reduced in intensity compared to C–C/C–H and O–C=O. This is likely to be due to the presence of O–C=O groups on the dyads at the binding positions. The XPS spectra of the ruthenium complexes show the peak for Ru 3d_5/2_ with a binding energy of 281.7 eV, thus supporting the presence of the Ru complex in the dyad. Meanwhile, Ru 3d_3/2_ peaks are hidden by the C 1s peak.^16,41^ The presence of the Ru complex is further supported by the N 1s peak shift to higher binding energy (core-level shown in Fig. S8, ESI†), which is more pronounced in the CBZ-3Py + Ru dyad (0.3 eV). Finally, the valence-band XPS (Fig. S9, ESI†) contains the overlapping O 2p and Ru 4d peaks. The intensity increase in the Ru 4d region (<3.5 eV) further confirms the presence of the coordination complex.

### Photoelectrochemical properties

**Fig. 4:**
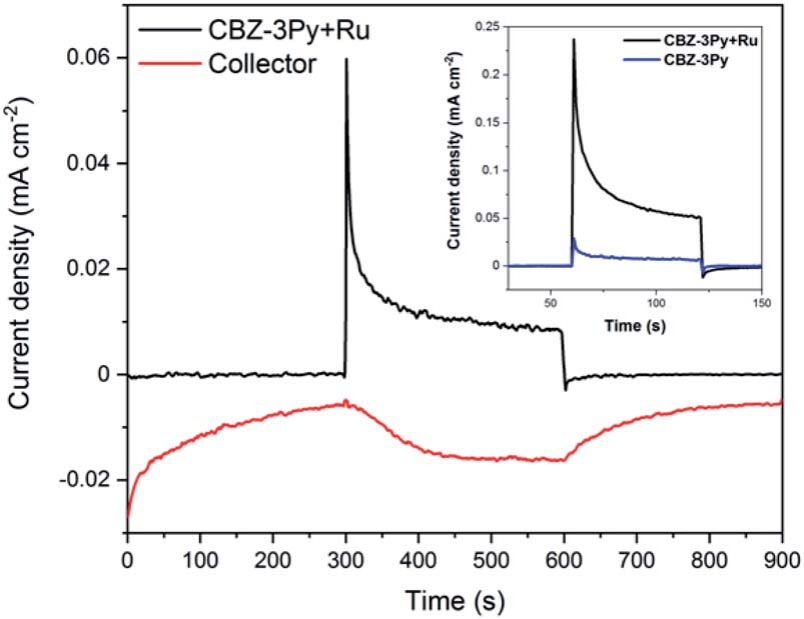
Collector–generator plot of a CBZ-3Py + Ru sensitized electrode. Black line: current–time trace at illuminated (200 W Xe lamp; 420 < *λ* < 800 nm) CBZ-3Py + Ru dyad on TiO_2_ in 0.1 M Na_2_SO_4_ at pH 5.8 with an applied bias of ∼0.5 V *versus* NHE. Red line: current–time traces at an FTO collector electrode, 400 μm from the photoanode at an applied bias of ∼−0.6 V *vs.* NHE measured concurrently with the photoelectrochemical–time trace (FE of shown measurement = 95%). Inset: 60 s chronoamperometry of CBZ-3Py (blue) and CBZ-3Py + Ru (black) in the same conditions of C-G measures.

The ability of the dyad-sensitized PECs to perform the photoinduced water splitting reaction was measured through photoelectrochemical measurements under white light illumination (200 W Xe lamp, 420 < *λ* < 800 nm). The bias applied was chosen according to the linear sweep voltammogram (LSV) of the sensitized TiO_2_ films (Fig. S10†) by selecting the potential at which the best light/dark ratio was recorded. The obtained photocurrents are shown in Fig. S11 and S12 (ESI),† and Fig. 4 (inset) for CBZ-4Py + Ru and CBZ-3Py + Ru sensitized electrodes, respectively. Each dyad-sensitized electrode has been checked against dye-sensitized electrodes (without WOC). In all cases, the dye-sensitized electrode was much less efficient compared to the dyad-sensitized electrode (Fig. S11 and S12, ESI,† and inset of Fig. 4). The CBZ-3Py dye-sensitized electrode had a negligible photocurrent of 3.5 μA cm^−2^, while the corresponding dyad CBZ-3Py + Ru, after a 10 min chronoamperometry, showed 25 μA cm^−2^, corresponding to an 8-fold increase. In the case of CBZ-4Py (8.6 μA cm^−2^) and the corresponding dyad CBZ-4Py + Ru (30 μA cm^−2^), a performance increase for the latter was observed, but was less pronounced (Fig. S12, ESI†). These values well match the behaviour of other organic sensitizers reported in the literature.^16,42,43^ Long-term measurements were performed to evaluate the stability of the dyad sensitized electrodes and are shown in ESI (Fig. S13 and S14†).

The O_2_ evolution was measured through the collector–generator technique,^38^ as recently reported by many studies on DS-PEC water splitting.^8,12,14,32,44^ In this technique, a second FTO working electrode (the collector) is positioned at a fixed distance (*ca.* 400 μm) from the photoanode (the generator) and kept at a reducing potential (−0.8 V *vs.* Ag/AgCl, ∼−0.6 V *vs.* NHE). In this way, the collector reduces the oxygen gas as soon as it is formed, thus transforming a very-difficult-to-evaluate variable (affected, among other things, by leakage and air contamination) into a simple electric signal. The direct quantification of oxygen production FE is possible from the ratio between the charge flowing through the collector and the one through the generator. The FE for CBZ-4Py + Ru and CBZ-3Py + Ru sensitized electrodes were 58 ± 12% and 88 ± 9% (average of 4 measurements on different samples), respectively (Fig. S15† and 4).

**Fig. 5:**
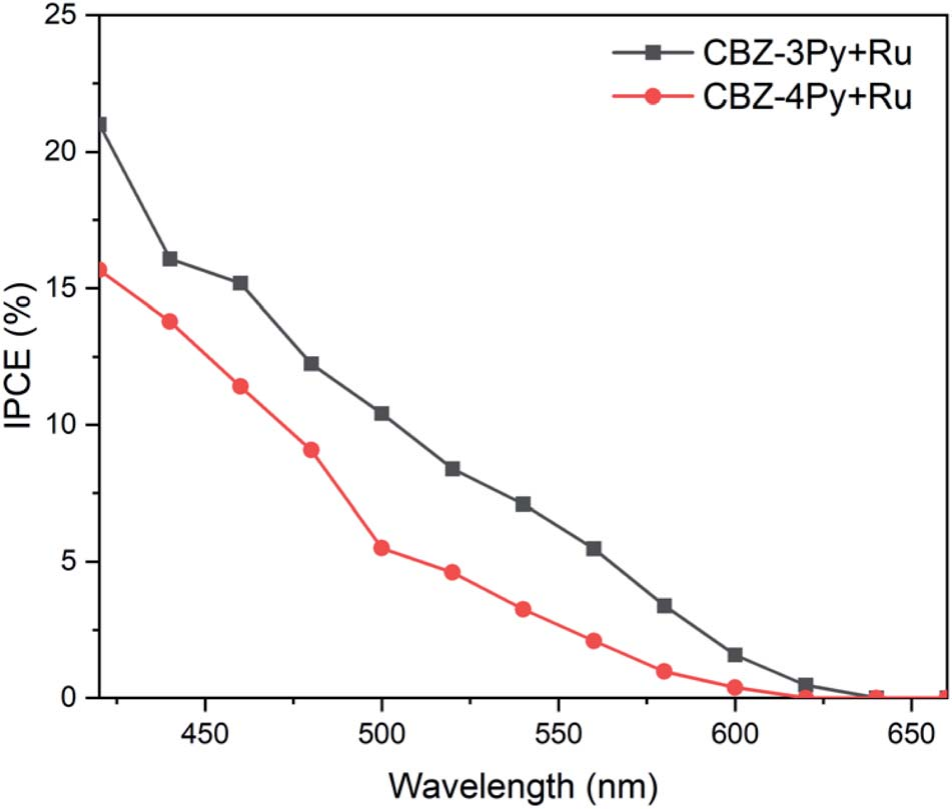
Incident photon-to-current efficiency (IPCE) of dyads sensitized TiO_2_ films in 0.1 M Na_2_SO_4_ at pH 5.8 with an applied bias of ∼0.5 V *vs.* NHE under monochromatic illumination.

To gain a better insight on the performances of the dyads to convert photons to electrons the incident photon-to-current efficiency (IPCE) was also measured (Fig. 5). The recorded IPCE well matched the trend of the measured faradaic efficiency. The intensity of the signal, related to the produced current density, was higher for the CBZ-3Py + Ru sensitized electrode, with a maximum efficiency of ∼16% at 450 nm. The IPCE of the TiO_2_ films sensitized with the dyads were compared with those of the corresponding films sensitized with the dye only (without WOC, Fig. S16 and S17†). The light harvesting efficiency (LHE) of both dyads sensitized electrodes were also measured to calculate the absorbed photon-to-current efficiency (APCE), shown in Fig. S18 and S19 (ESI).†

It is worth noting, as an addition evidence of the formation of the dyads, that the IPCE of the devices sensitized with the dye by itself (*e.g.*, without the integrated catalyst) showed a negligible efficiency, in agreement with the hereabove described photocurrent experiments (see Fig. S11 and S12 in ESI† and inset of Fig. 4).

The notably higher efficiency of the 3Py derivative, compared with the 4Py analogue, can be ascribed to the fact that in the former dyad the rotation about the single bond (between the methylene bridge and the quaternary carbon atom of pyridine) allows the catalyst portion to come closer to the dye. Therefore, a more efficient charge transfer between the dye and the catalyst fragment of the dyad is ensured (see the simulated movie in ESI†).

## Conclusions

In conclusion, a new series of metal-free organic dyes, bearing a coordinating pyridine functionality, has been synthesized, fully characterised and used to sensitize photoanodes for photoelectrochemical water oxidation. These sensitizers have been used to coordinate a proper Ru precursor to yield the first example ever of a molecular dyad based on a metal-free organic dye integrated to a benchmark Ru complex for water oxidation. Optical, electrochemical, and XPS experiments confirmed the formation of the dyad and, in particular, the presence of the Ru catalyst anchored to the TiO_2_ photoanode *via* the dye linker. Both dyad-sensitized photoanodes showed state-of-the-art FE in the O_2_ generation process,^8,45–47^ with the CBZ-3Py + Ru dyad reaching an average efficiency of nearly 90% and a best cell efficiency of 95%. Moreover, IPCE measurements confirmed the validity of the approach showing efficiencies among the best recorded for similar compounds and noticeably supported the evidence of the formation of dyads when compared to isolated dyes. Further studies, including a detailed computational investigation, are currently underway to further support and extend the potential of the herein introduced molecular dyad approach to water splitting.

## Conflicts of interest

There are no conflicts to declare.

## Supplementary Material

RA-011-D0RA10971A-s001

RA-011-D0RA10971A-s002
