# Supplementary material for: Dye–catalyst dyads for photoelectrochemical water oxidation based on metal-free sensitizers
Source: RSC Adv. 2021 Jan 28;11(10):5311–9. doi: 10.1039/d0ra10971a (PMC8694650; doi:10.1039/d0ra10971a)
Supplement: RA-011-D0RA10971A-s002 [file RA-011-D0RA10971A-s002.pdf]

Electronic Supplementary Material (ESI) for RSC Advances.
This journal is © The Royal Society of Chemistry 2021

Electronic Supplementary Information

Dye-catalyst dyads for photoelectrochemical water oxidation based on metal-free sensitizers

Cristina Decavoli,[a] Chiara L. Boldrini,[a] Vanira Trifiletti,[b] Sally Luong,[b] Oliver Fenwick,[b] Norberto Manfredi,*[a] and Alessandro Abbotto*[a]

[a] Department of Materials Science and INSTM Unit, Solar Energy Research Center MIB-SOLAR, University of Milano – Bicocca, Via R. Cozzi 55, I-20125, Milano, Italy

[b] School of Engineering and Materials Science (SEMS), Queen Mary University of London, Mile End Road, London E1 4NS, UK

Email: alessandro.abbotto@unimib.it; norberto.manfredi@unimib.it

Table of Content

Figure S1. Absorption spectra of 1-µm TiO_2 film sensitized by the investigated dyes (solid line) and corresponding dyads (dash-dot line)....4

Figure S2. CV of dyes in solution in $TBAClO_4$ 0.1 M in DMF degassed with Ar....4

Figure S3. DPV of dyes in solution in $TBAClO_4$ 0.1 M in DMF degassed with Ar....5

Figure S4. CV of dye CBZ-4Py, corresponding dyad CBZ-4Py+Ru, and of the Ru-precursor (Ru) on a 3-µm-thick TiO_2 film (0.196 cm^2) in $TBAClO_4$ 0.1 M in CH_3CN degassed with Ar. 5

Figure S5. CV of dye CBZ-3Py, corresponding dyad CBZ-3Py+Ru, and of the Ru-precursor (Ru) on a 3-µm-thick TiO_2 film (0.196 cm^2) in $TBAClO_4$ 0.1 M in CH_3CN degassed with Ar. 6

Figure S6. CV of CBZ-4Py, CBZ-4Py+Ru and corresponding N-alkylcarbazole (CBZ-Th) on 3.5-µm-thick TiO_2 film in $TBAClO_4$ 0.1 M in CH_3CN degassed with Ar....6

Figure S7. XPS survey spectra of the investigated dyes and dyads compared to the TiO_2 spectrum; core-levels involved in the bonding interaction between TiO_2 and the dyes are labelled in red....7

Figure S8. XPS spectra of the high-resolution core level for N 1s region of the investigated dyes and dyads compared to the bare TiO_2....7

Figure S9. XPS valence-level spectrum of the investigated dyes and dyads compared to the bare TiO_2; in the inset, the enlargement of the spectrum in the region where the contribution of ruthenium is clearly visible below 3.5 eV....8

Figure S10. Linear sweep voltammogram (LSV) of TiO_2 films sensitized with CBZ-4Py+Ru (red) and CBZ-3Py+Ru (black) in 0.1 M Na_2SO_4 at pH 5.8 under chopped illumination (200 W Xe lamp; 420<λ<800 nm)....8

Figure S11. Chronoamperometry of TiO_2 films sensitized with CBZ-3Py (red) and CBZ-3Py+Ru (black) in 0.1 M Na_2SO_4 at pH 5.8 with an applied bias of ~0.5 V vs NHE under illumination (200 W Xe lamp; 420<λ<800 nm)....9

Figure S12. Chronoamperometry of TiO_2 films sensitized with CBZ-4Py (red) and CBZ-4Py+Ru (black) in 0.1 M Na_2SO_4 at pH 5.8 with an applied bias of ~0.5 V vs NHE under illumination (200 W Xe lamp; 420<λ<800 nm)....9

Figure S13. 2-hours-chronoamperometry of TiO_2 films sensitized with CBZ-3Py+Ru in 0.1 M Na_2SO_4 at pH 5.8 with an applied bias of ~0.5 V vs NHE under illumination (200 W Xe lamp; 420<λ<800 nm)....10

Figure S14. 2-hours-chronoamperometry of TiO_2 films sensitized with CBZ-4Py+Ru in 0.1 M Na_2SO_4 at pH 5.8 with an applied bias of ~0.5 V vs NHE under illumination (200 W Xe lamp; 420<λ<800 nm)....10

Figure S15. Collector-Generator plot of a CBZ-4Py+Ru sensitized electrode. Black line: current–time trace at illuminated (200 W Xe lamp; 420<λ<800 nm) CBZ-4Py+Ru dyad on TiO_2 in 0.1 M Na_2SO_4 at pH 5.8 with an applied bias of ~0.5 V vs NHE. Red line: current–time traces at an FTO collector electrode, 300 µm from the photoanode at an applied bias of ~ -0.6 V vs NHE measured concurrently with the photoelectrochemical–time trace (FE of shown measurement = 65%)....11

Figure S16. Incident photon-to-current efficiency (IPCE) of TiO_2 films sensitized with CBZ-3Py (black) and CBZ-3Py+Ru (red) in 0.1 M Na_2SO_4 at pH 5.8 with an applied bias of ~0.5 V vs NHE under monochromatic illumination. 11

Figure S17. Incident photon to current efficiency of TiO_2 films sensitized with CBZ-4Py (black) and CBZ-4Py+Ru (red) in 0.1 M Na_2SO_4 at pH 5.8 with an applied bias of ~0.5 V vs NHE under monochromatic illumination. 12

Figure S18. Light harvesting efficiency (LHE) of TiO_2 films sensitized with the investigated dyads. 12

Figure S19. Absorbed photon-to-current efficiency (APCE) of TiO_2 films sensitized with the investigated dyads. 13

^1H and ^{13}C NMR *14*

Figure S20. ^1H NMR of 2 in $CDCl_3$. 14

Figure S21. ^1H NMR of 3a in $CDCl_3$. 14

Figure S22. ^1H NMR of 3b in $CDCl_3$. 15

Figure S23. ^1H NMR of 4a in DMSO-d_6. 15

Figure S24. ^1H NMR of 4b in DMSO-d_6. 16

Figure S25. ^1H NMR of CBZ-4Py in DMSO-d_6. 16

Figure S26. ^{13}C NMR of CBZ-4Py in DMSO-d_6. 17

Figure S27. ^1H NMR of CBZ-3Py in DMSO-d_6. 17

Figure S28. ^{13}C NMR of CBZ-3Py in DMSO-d_6. 18

IR spectra *19*

Figure S29: FT-IR spectrum of CBZ-4Py 19

Figure S30: FT-IR spectrum of CBZ-3Py 19

HRMS spectra *20*

Figure S31. HRMS spectrum of CBZ-4Py. 20

Figure S32. HRMS spectrum of CBZ-3Py. 20

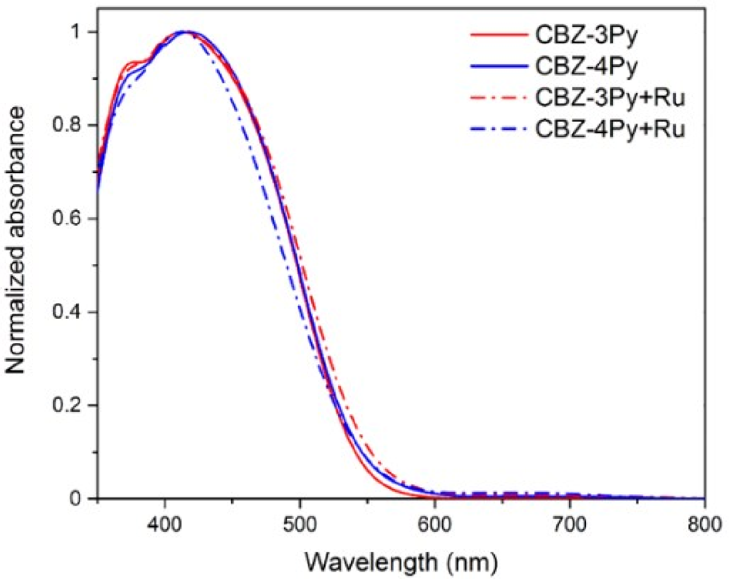

Figure S1. *Absorption spectra of 1-μm TiO_2 film sensitized by the investigated dyes (solid line) and corresponding dyads (dash-dot line).*

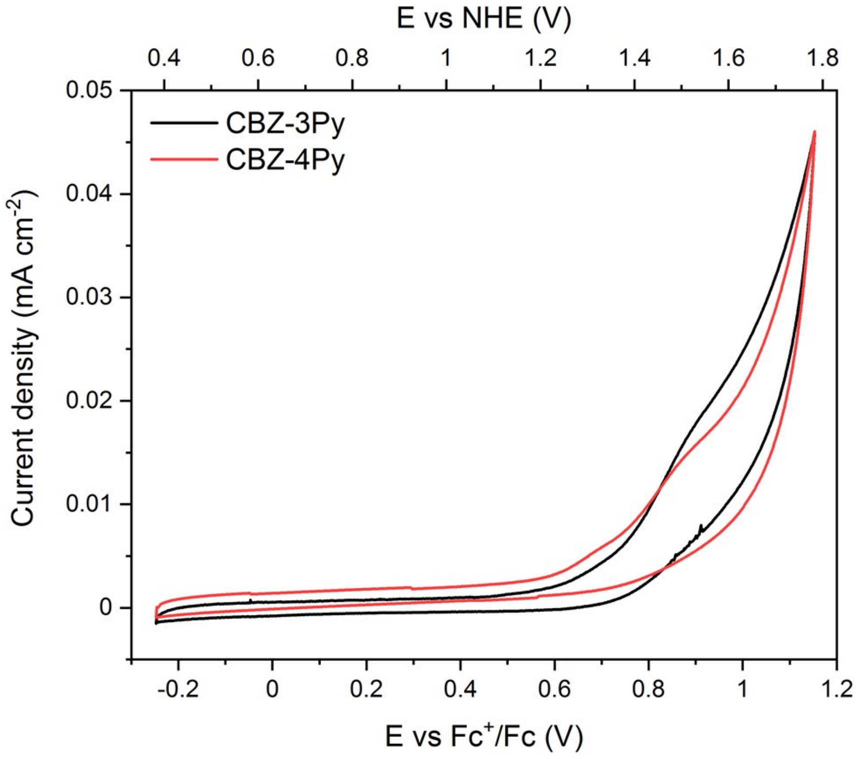

Figure S2. *CV of dyes in solution in $TBAClO_4$ 0.1 M in DMF degassed with Ar.*

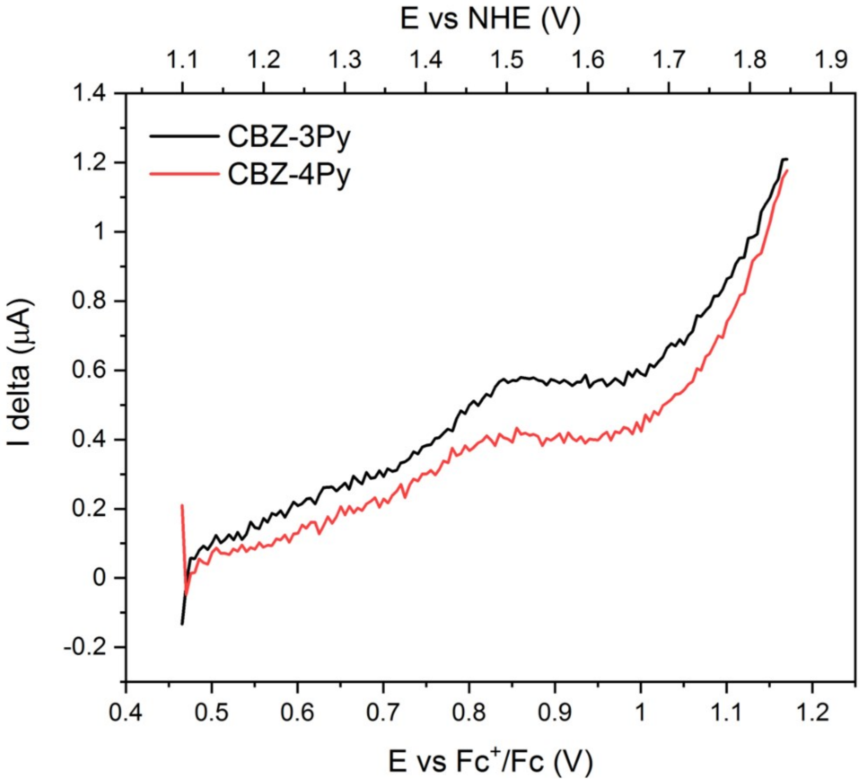

Figure S3. *DPV of dyes in solution in $TBAClO_4$ 0.1 M in DMF degassed with Ar.*

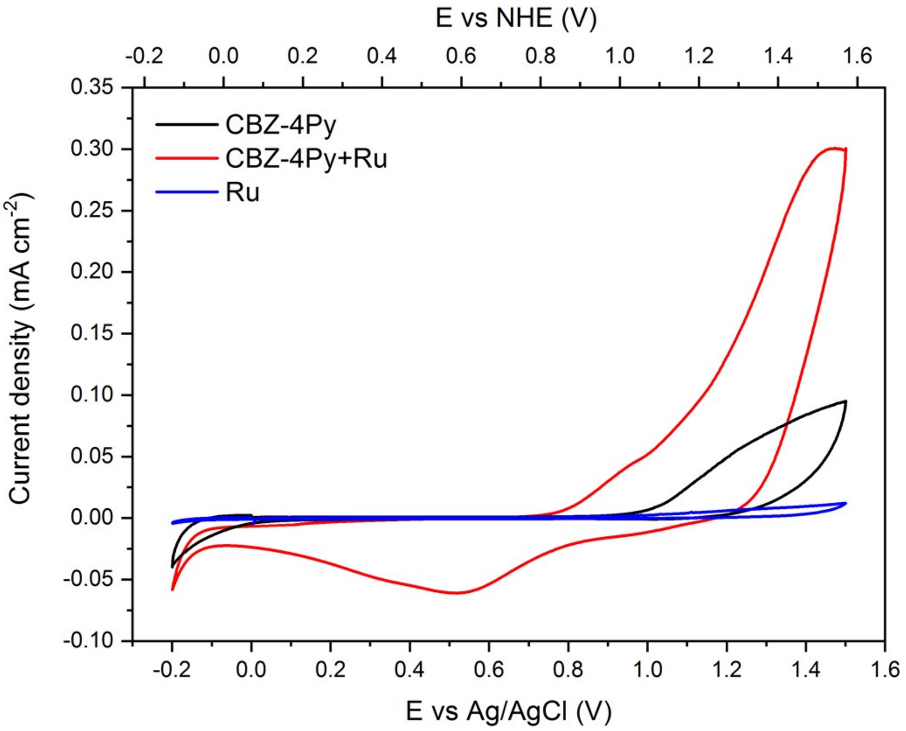

Figure S4*. CV of dye* ***CBZ-4Py****, corresponding dyad* ***CBZ-4Py+Ru****, and of the Ru-precursor (Ru) on a 3-μm-thick TiO_2 film (0.196 cm^2) in $TBAClO_4$ 0.1 M in CH_3CN degassed with Ar.*

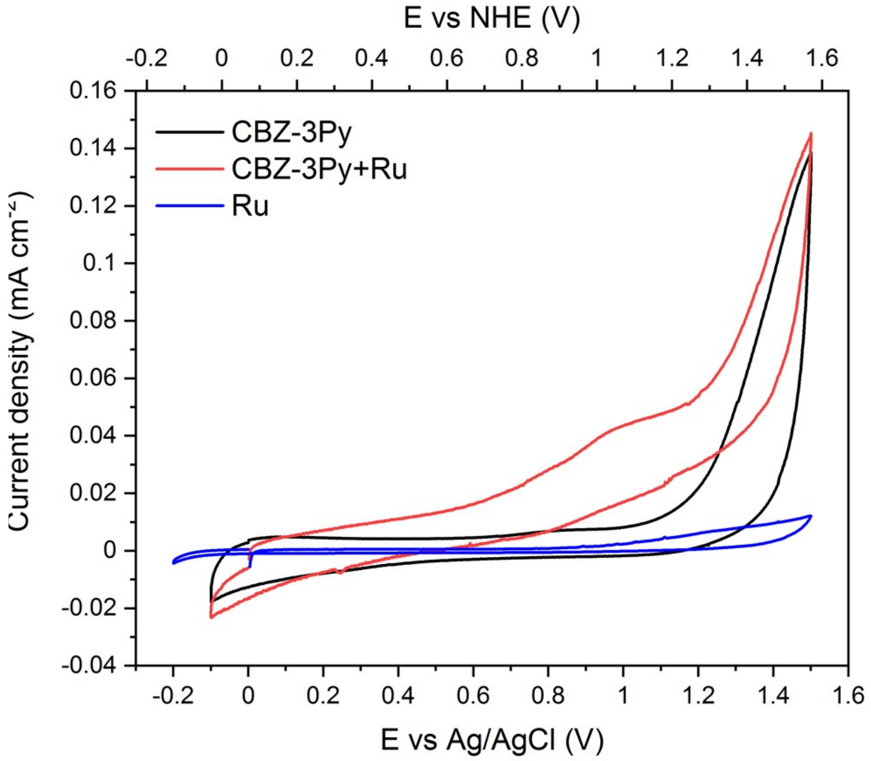

Figure S5. *CV of dye **CBZ-3Py**, corresponding dyad **CBZ-3Py+Ru**, and of the Ru-precursor (Ru) on a 3-µm-thick TiO_2 film (0.196 cm^2) in $TBAClO_4$ 0.1 M in CH_3CN degassed with Ar.*

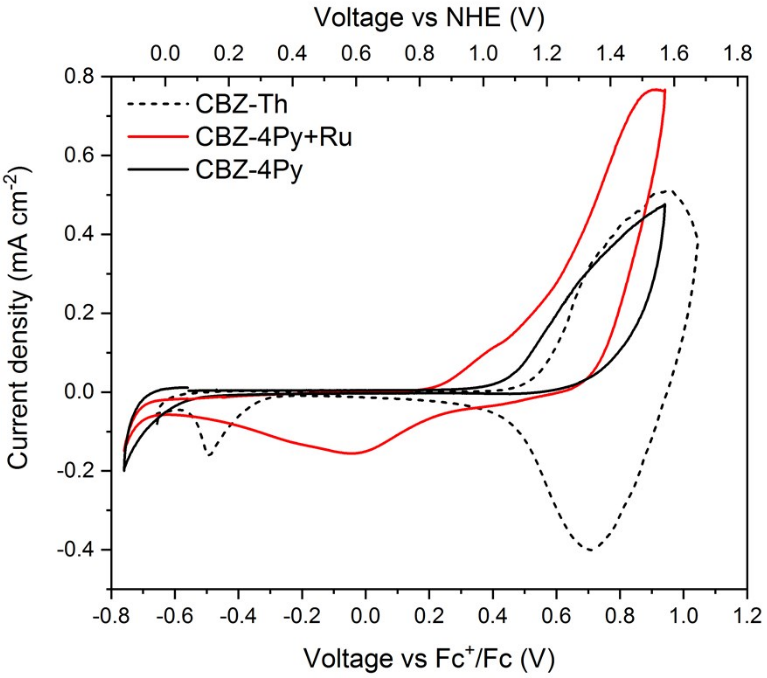

Figure S6. *CV of **CBZ-4Py**, **CBZ-4Py+Ru** and corresponding N-alkylcarbazole (**CBZ-Th**) on 3.5-µm-thick TiO_2 film in $TBAClO_4$ 0.1 M in CH_3CN degassed with Ar.*

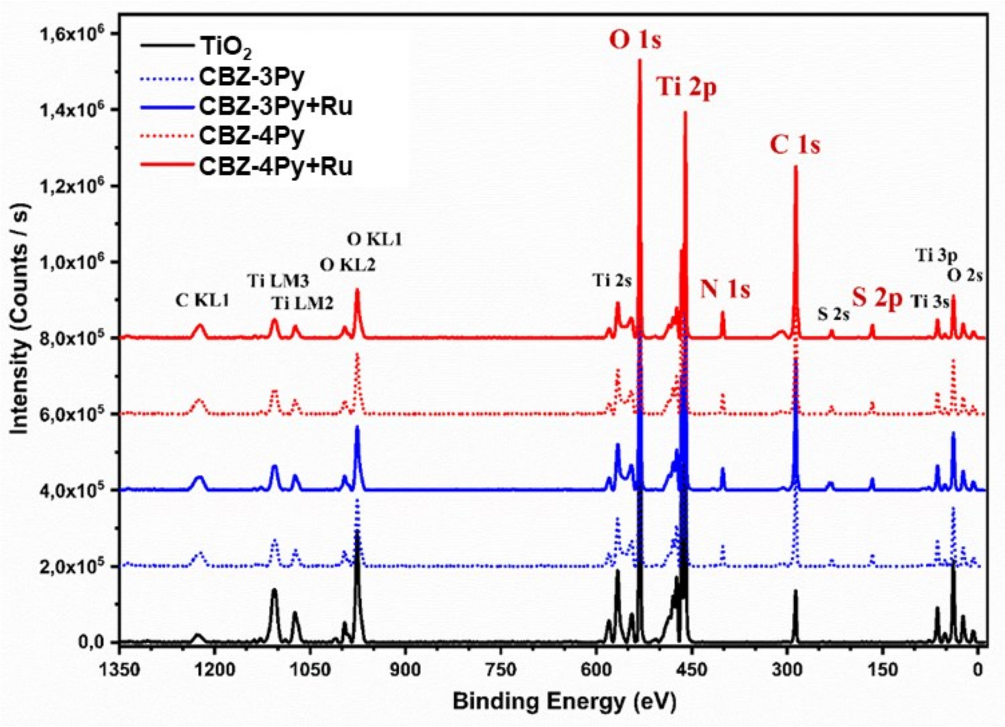

Figure S7. *XPS survey spectra of the investigated dyes and dyads compared to the TiO_2 spectrum; core-levels involved in the bonding interaction between TiO_2 and the dyes are labelled in red.*

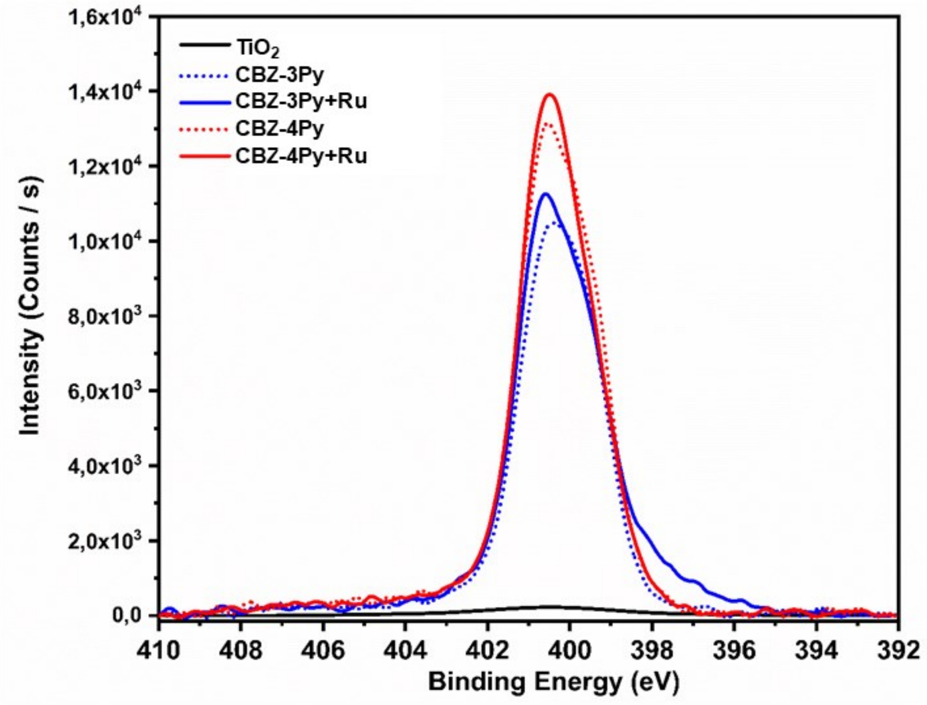

Figure S8. *XPS spectra of the high-resolution core level for N 1s region of the investigated dyes and dyads compared to the bare TiO_2.*

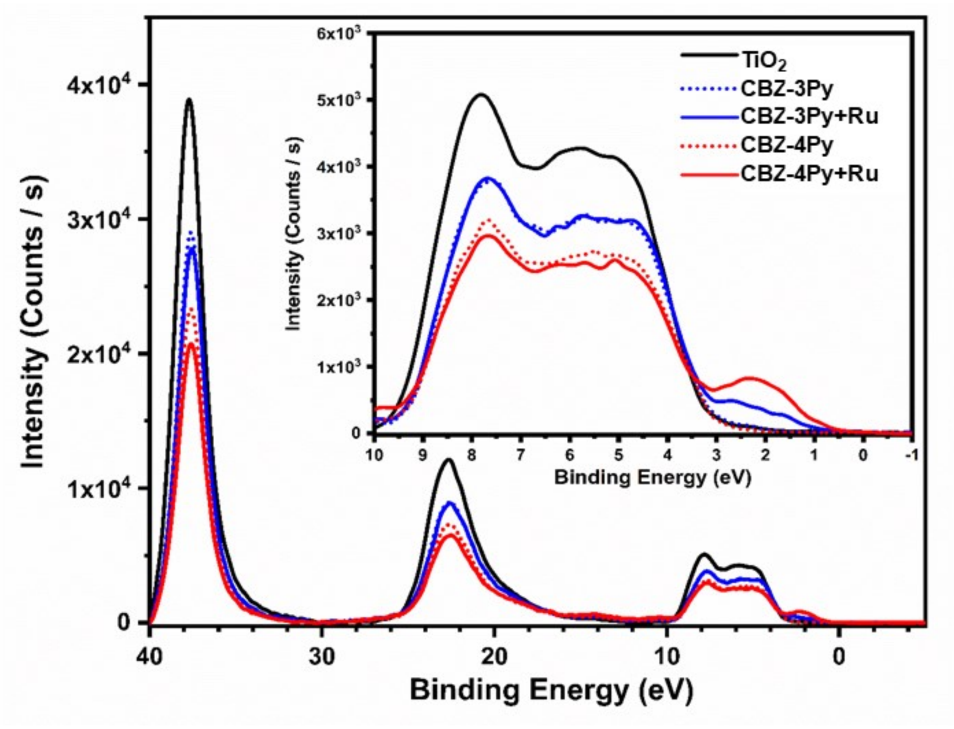

Figure S9. *XPS valence-level spectrum of the investigated dyes and dyads compared to the bare TiO_2; in the inset, the enlargement of the spectrum in the region where the contribution of ruthenium is clearly visible below 3.5 eV.*

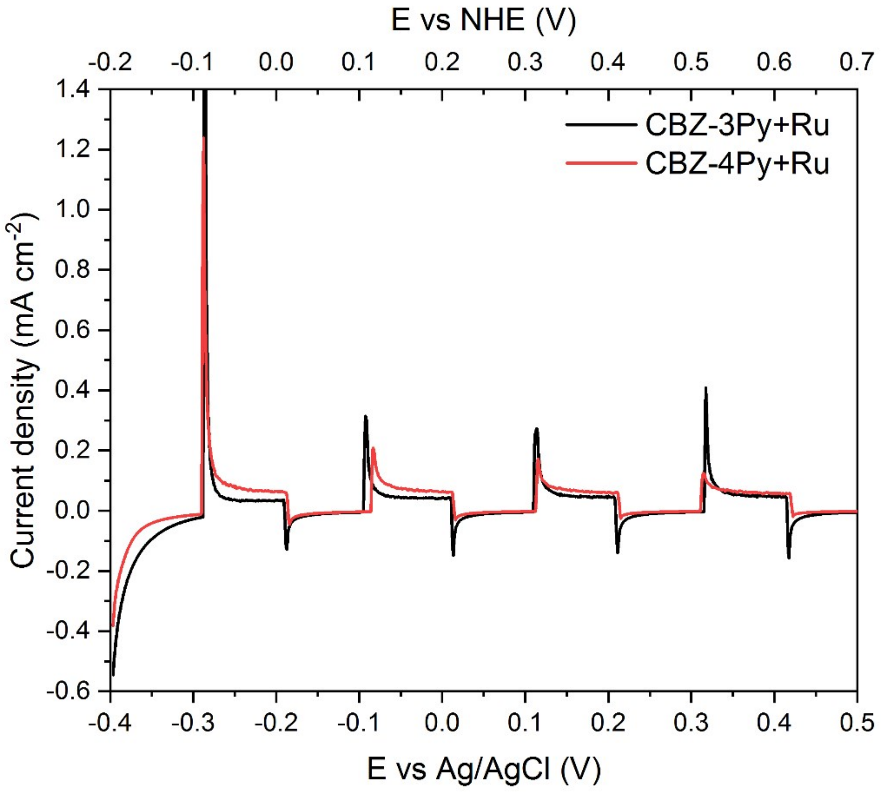

Figure S10. *Linear sweep voltammogram (LSV) of TiO_2 films sensitized with **CBZ-4Py+Ru** (red) and **CBZ-3Py+Ru** (black) in 0.1 M Na_2SO_4 at pH 5.8 under chopped illumination (200 W Xe lamp; 420<λ<800 nm)*

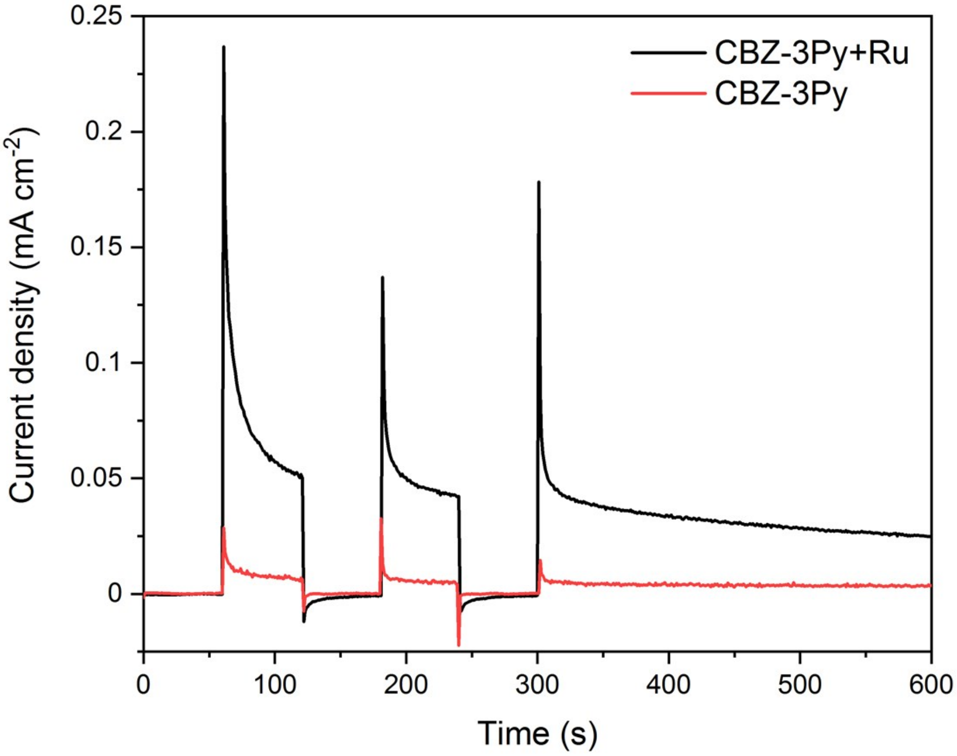

Figure S11. *Chronoamperometry of TiO_2 films sensitized with* ***CBZ-3Py*** *(red) and* ***CBZ-3Py+Ru*** *(black) in 0.1 M Na_2SO_4 at pH 5.8 with an applied bias of ~0.5 V vs NHE under illumination (200 W Xe lamp; $420<\lambda<800$ nm).*

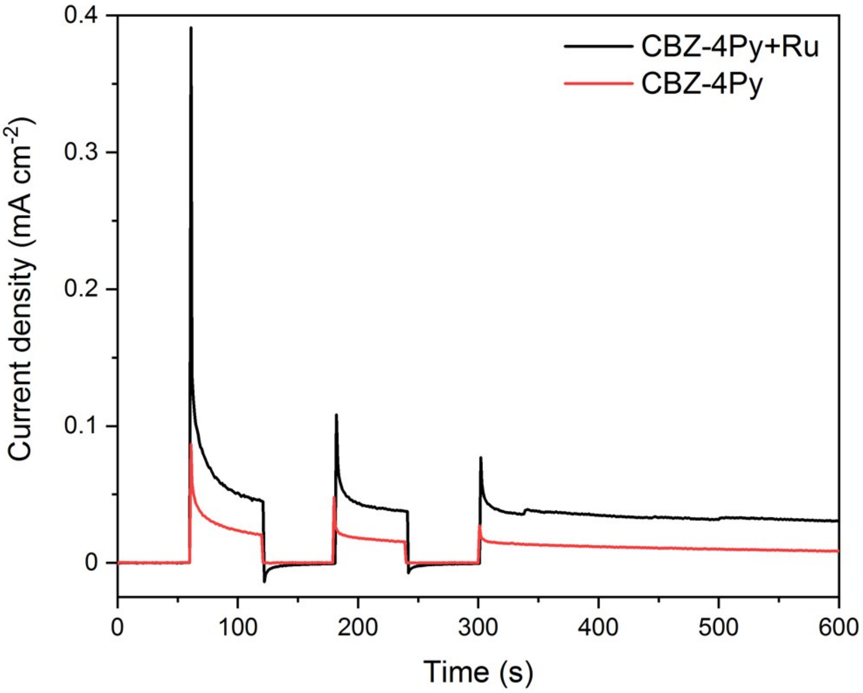

Figure S12. *Chronoamperometry of TiO_2 films sensitized with* ***CBZ-4Py*** *(red) and* ***CBZ-4Py+Ru*** *(black) in 0.1 M Na_2SO_4 at pH 5.8 with an applied bias of ~0.5 V vs NHE under illumination (200 W Xe lamp; $420<\lambda<800$ nm).*

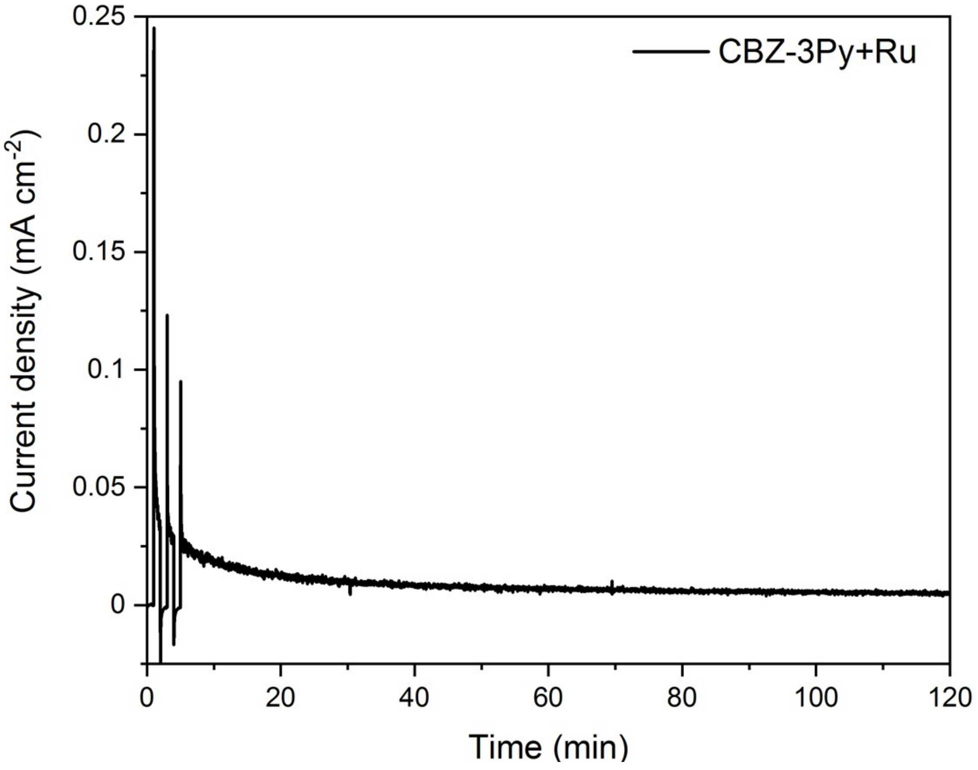

Figure S13. *2-hours-chronoamperometry of TiO_2 films sensitized with* ***CBZ-3Py+Ru*** *in 0.1 M Na_2SO_4 at pH 5.8 with an applied bias of ~0.5 V vs NHE under illumination (200 W Xe lamp; 420<λ<800 nm).*

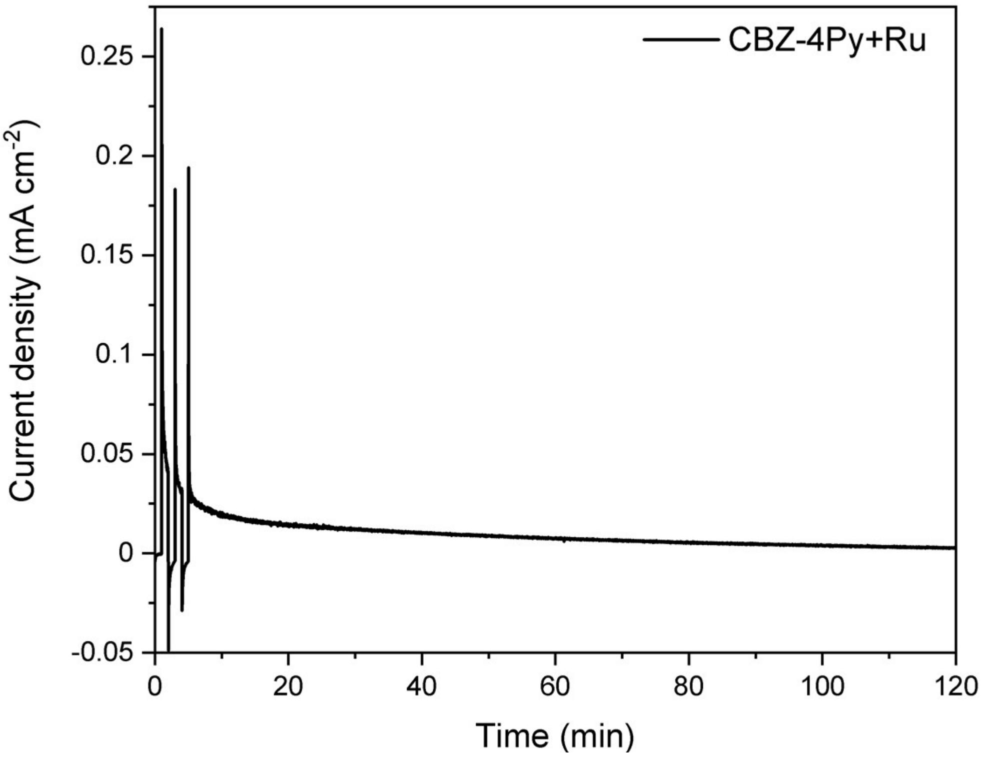

Figure S14. *2-hours-chronoamperometry of TiO_2 films sensitized with* ***CBZ-4Py+Ru*** *in 0.1 M Na_2SO_4 at pH 5.8 with an applied bias of ~0.5 V vs NHE under illumination (200 W Xe lamp; 420<λ<800 nm).*

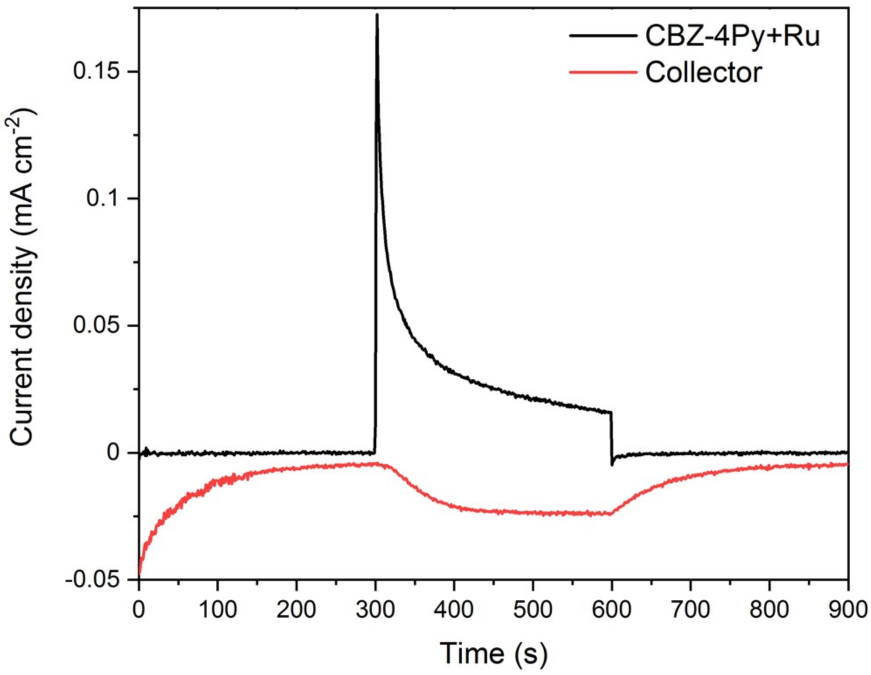

Figure S15. *Collector-Generator plot of a* ***CBZ-4Py+Ru*** *sensitized electrode. Black line: current–time trace at illuminated (200 W Xe lamp; 420<λ<800 nm)* ***CBZ-4Py+Ru*** *dyad on TiO_2 in 0.1 M Na_2SO_4 at pH 5.8 with an applied bias of ~0.5 V vs NHE. Red line: current–time traces at an FTO collector electrode, 300 µm from the photoanode at an applied bias of ~ -0.6 V vs NHE measured concurrently with the photoelectrochemical–time trace (FE of shown measurement = 65%).*

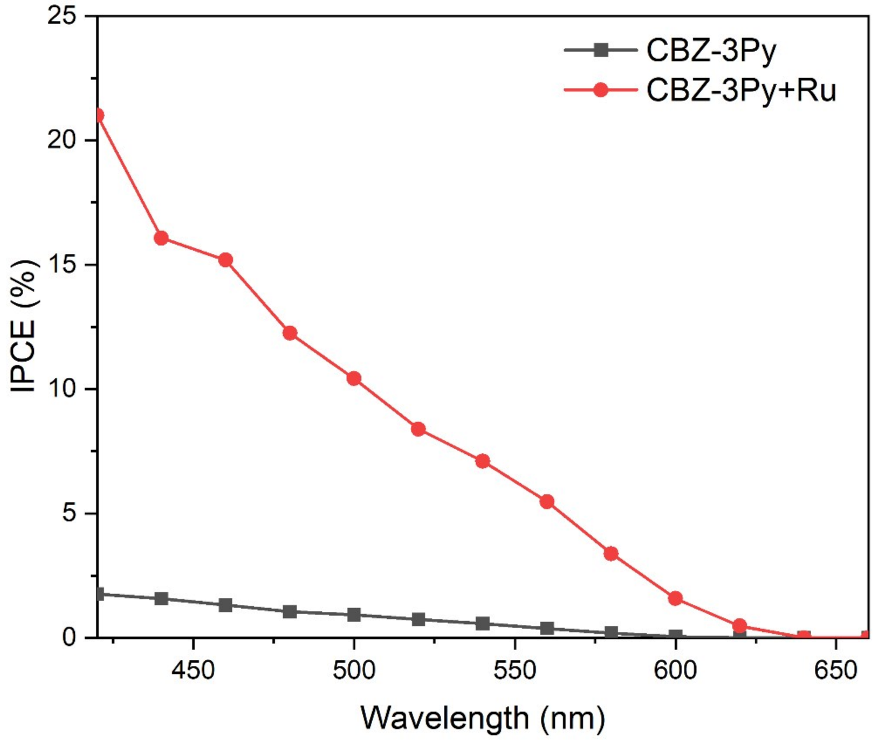

Figure S16*. Incident photon-to-current efficiency (IPCE) of TiO_2 films sensitized with* ***CBZ-3Py*** *(black) and* ***CBZ-3Py+Ru*** *(red) in 0.1 M Na_2SO_4 at pH 5.8 with an applied bias of ~0.5 V vs NHE under monochromatic illumination.*

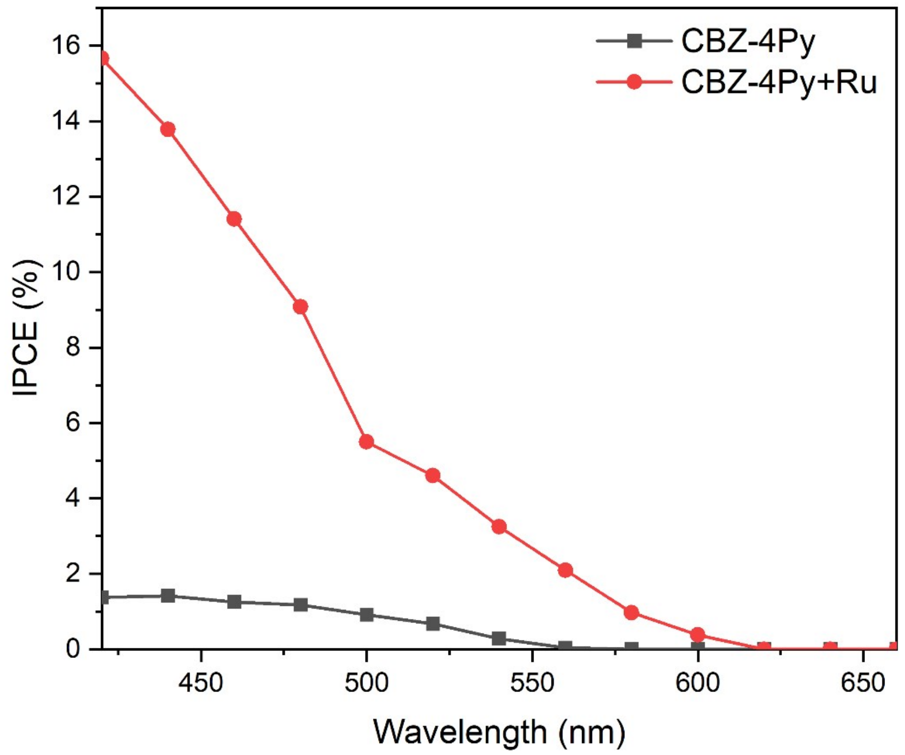

Figure S17. *Incident photon to current efficiency of TiO_2 films sensitized with* ***CBZ-4Py*** *(black) and* ***CBZ-4Py+Ru*** *(red) in 0.1 M Na_2SO_4 at pH 5.8 with an applied bias of ~0.5 V vs NHE under monochromatic illumination.*

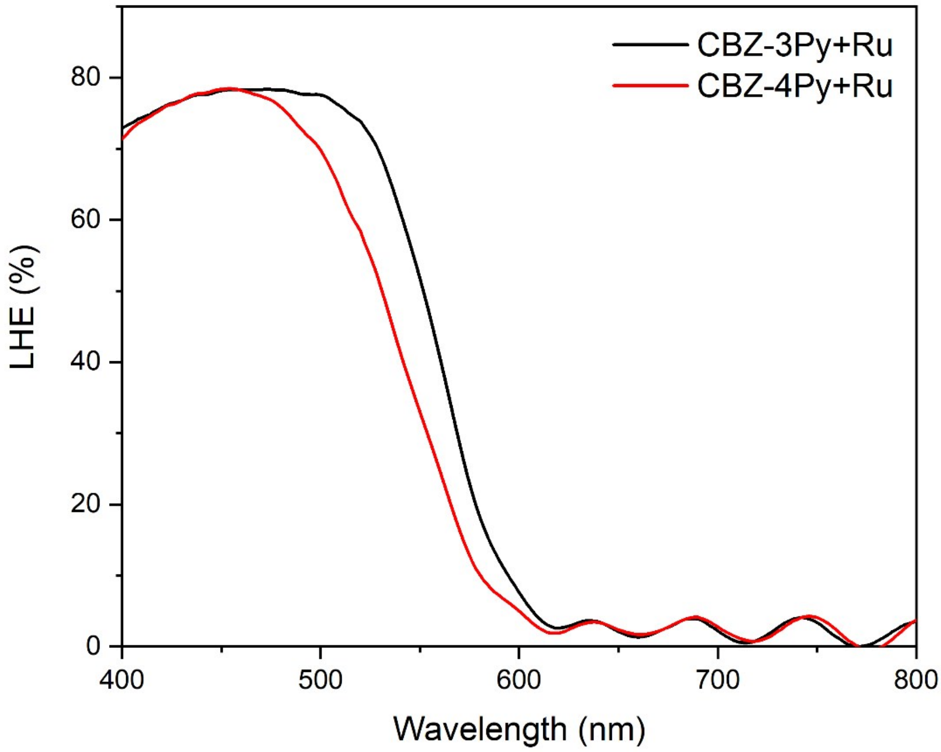

Figure S18. *Light harvesting efficiency (LHE) of TiO_2 films sensitized with the investigated dyads.*

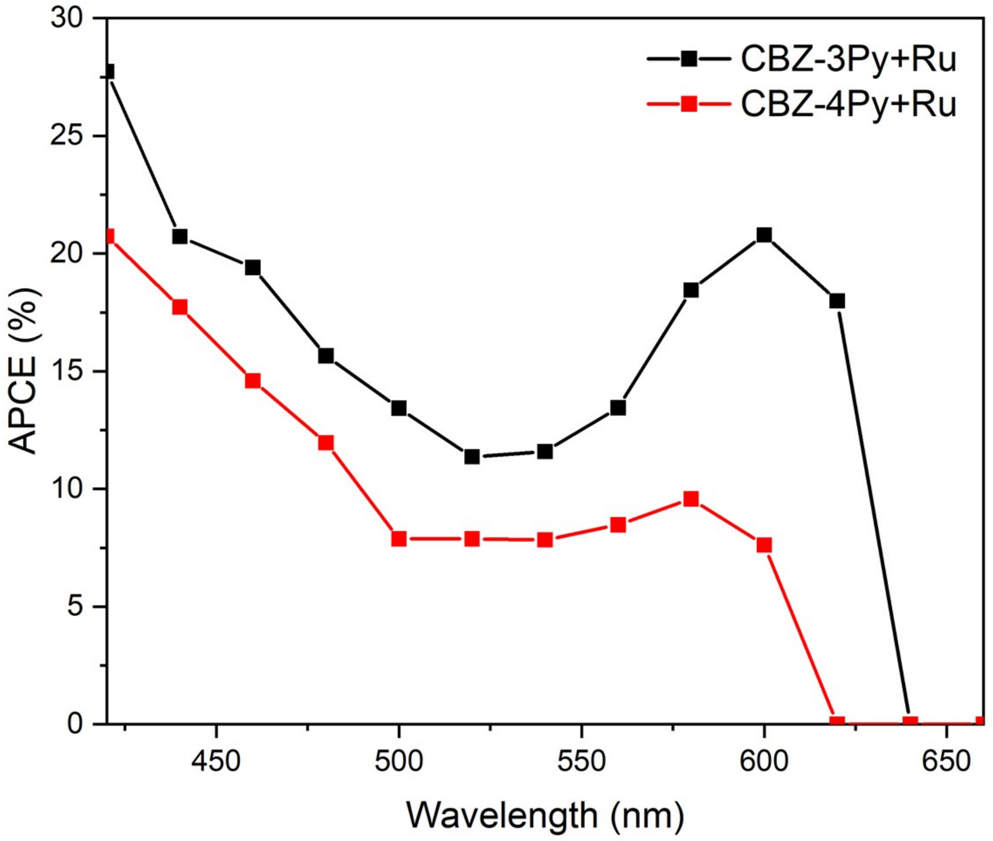

Figure S19*. Absorbed photon-to-current efficiency (APCE) of TiO_2 films sensitized with the investigated dyads.*

^{1}H and ^{13}C NMR

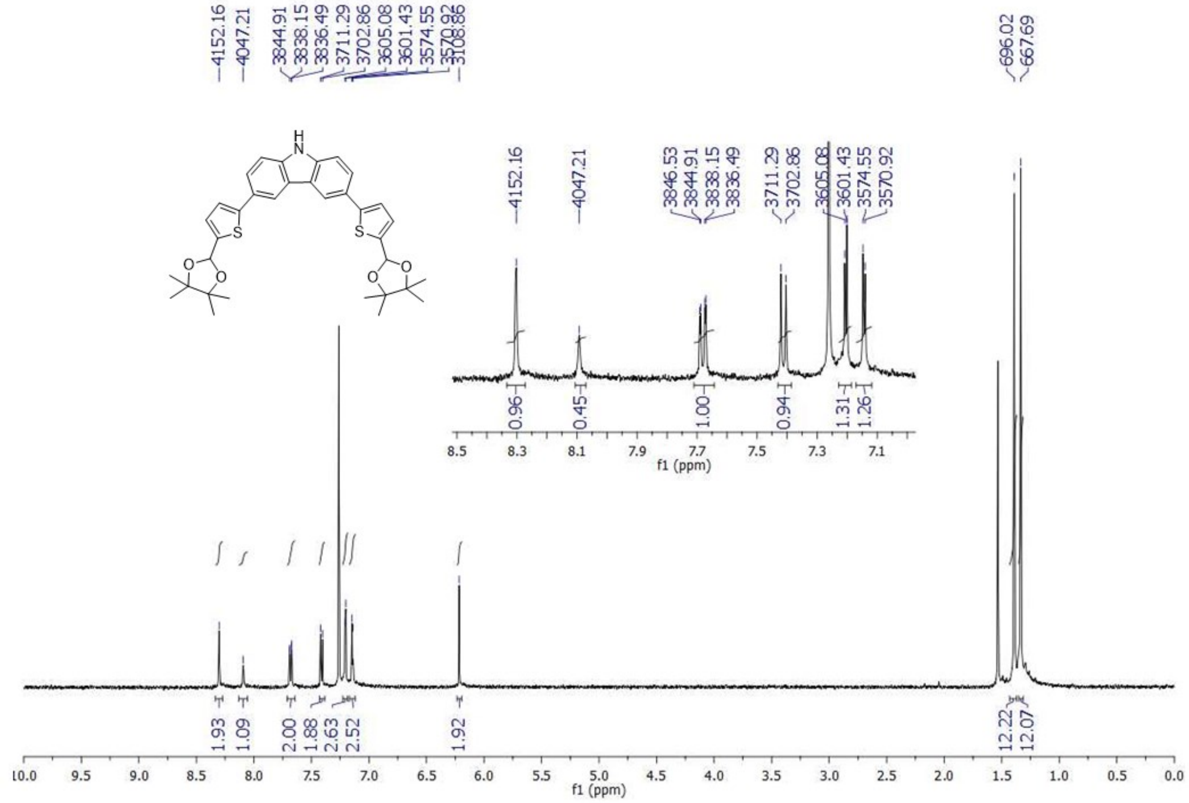

Figure S20. *^{1}H NMR of **2** in $CDCl_3$.*

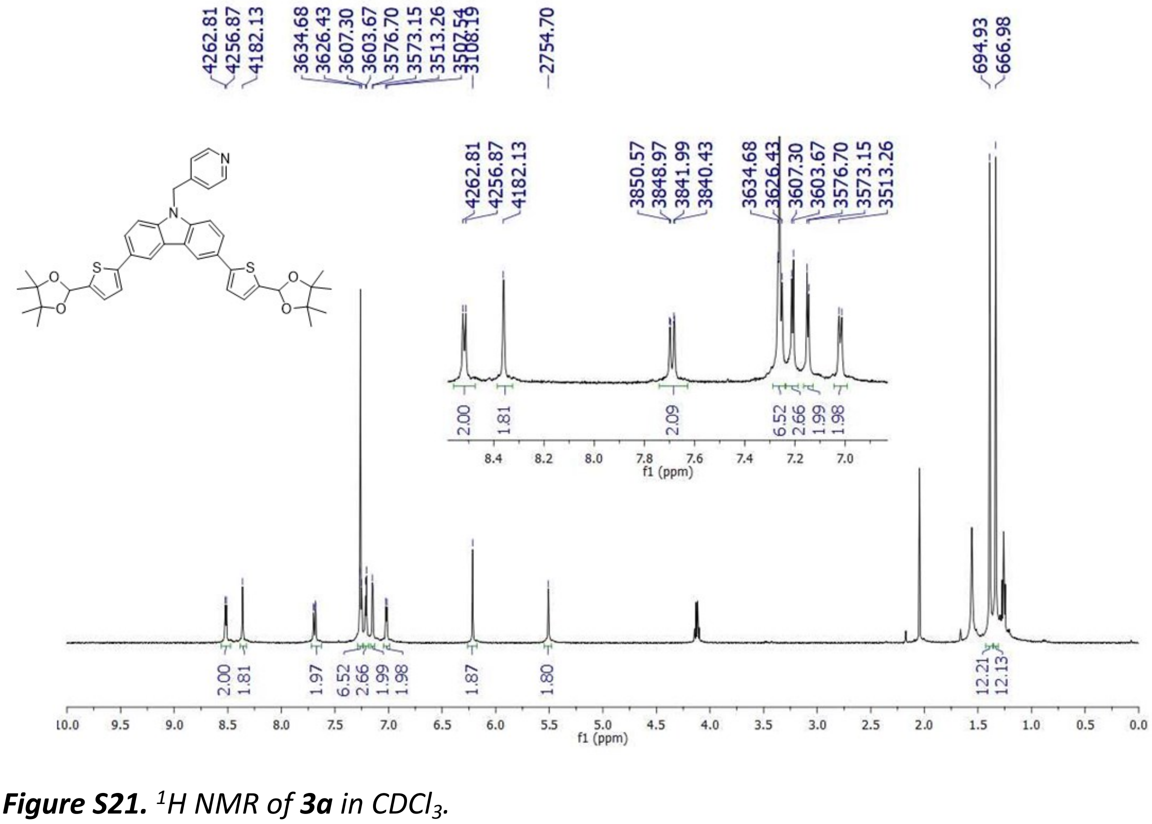

Figure S21. *^{1}H NMR of **3a** in $CDCl_3$.*

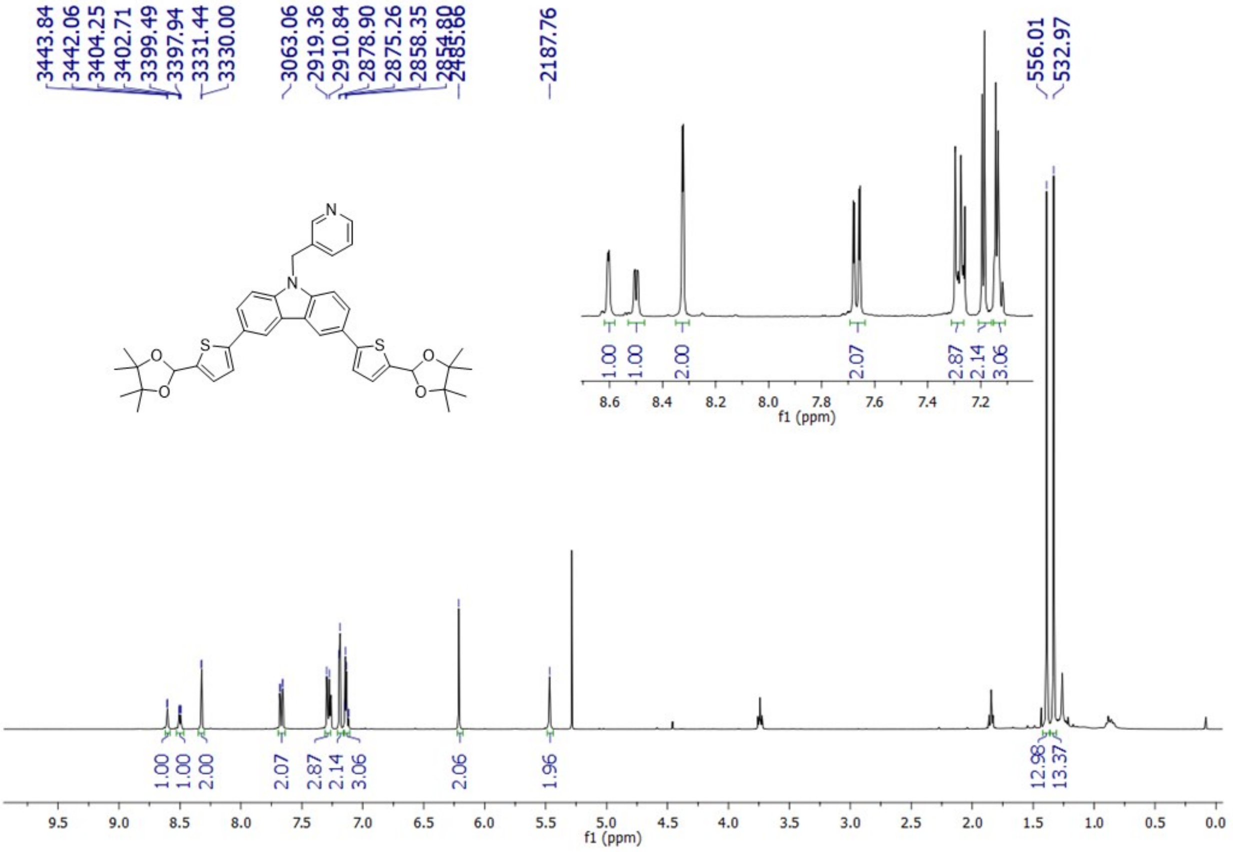

Figure S22. ^{1}H *NMR of **3b** in* $CDCl_3$*.*

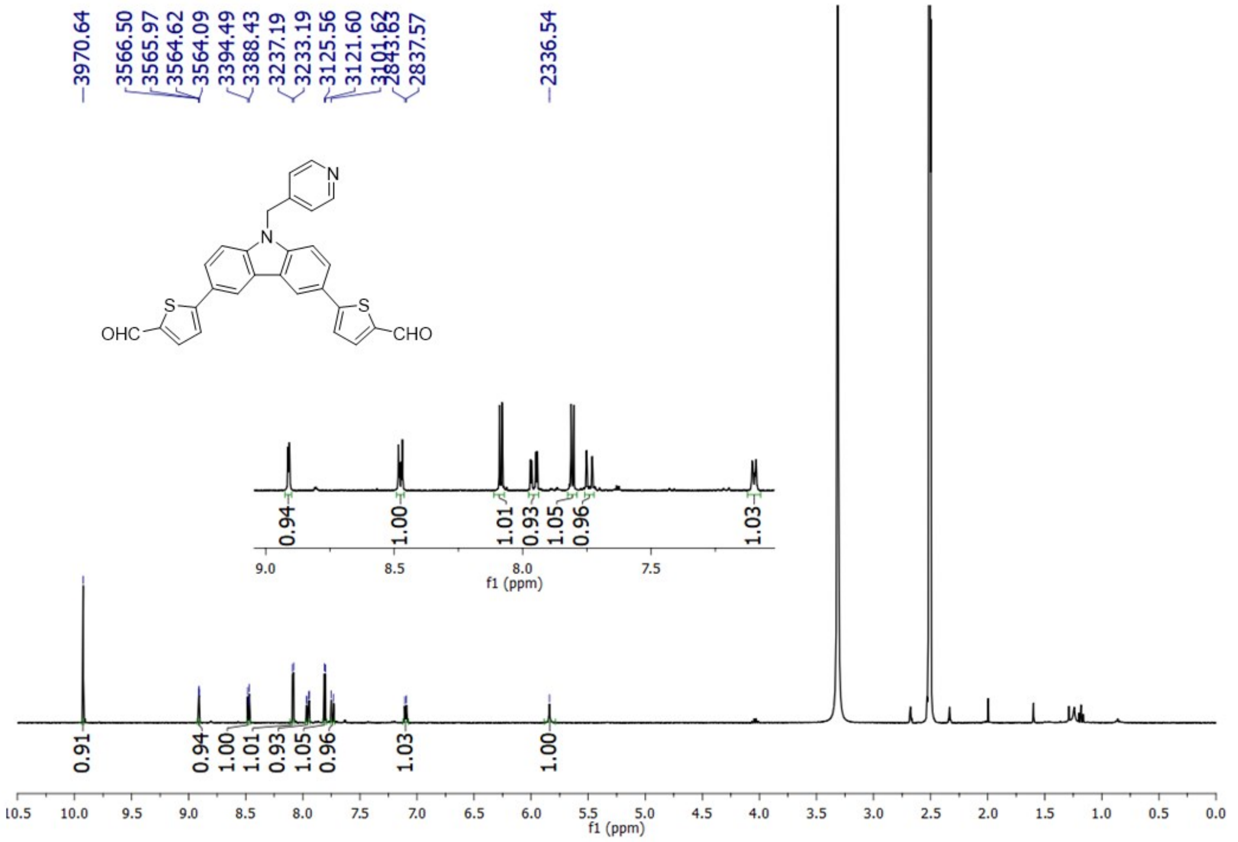

Figure S23. ^{1}H *NMR of **4a** in DMSO-*d_6*.*

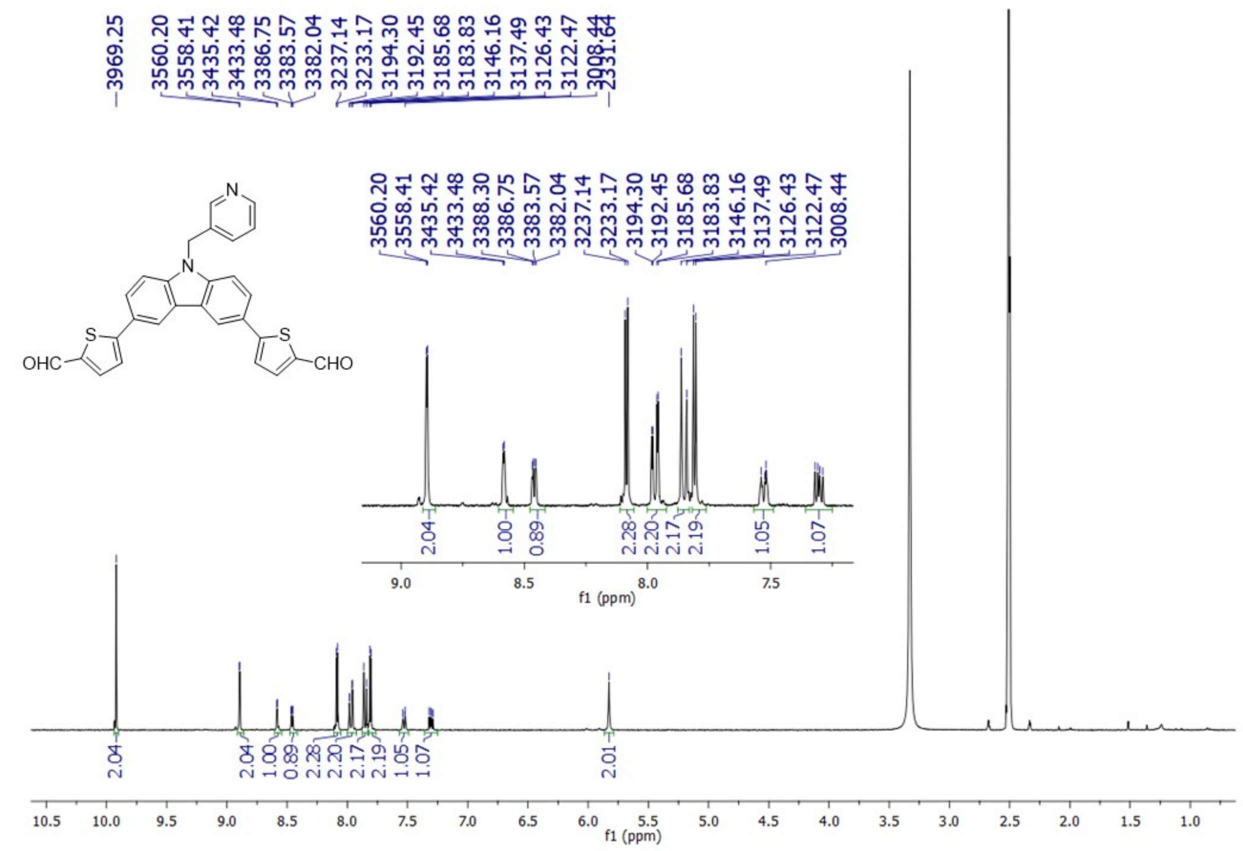

Figure S24. *^{1}H NMR of* ***4b*** *in DMSO-d$_6$.*

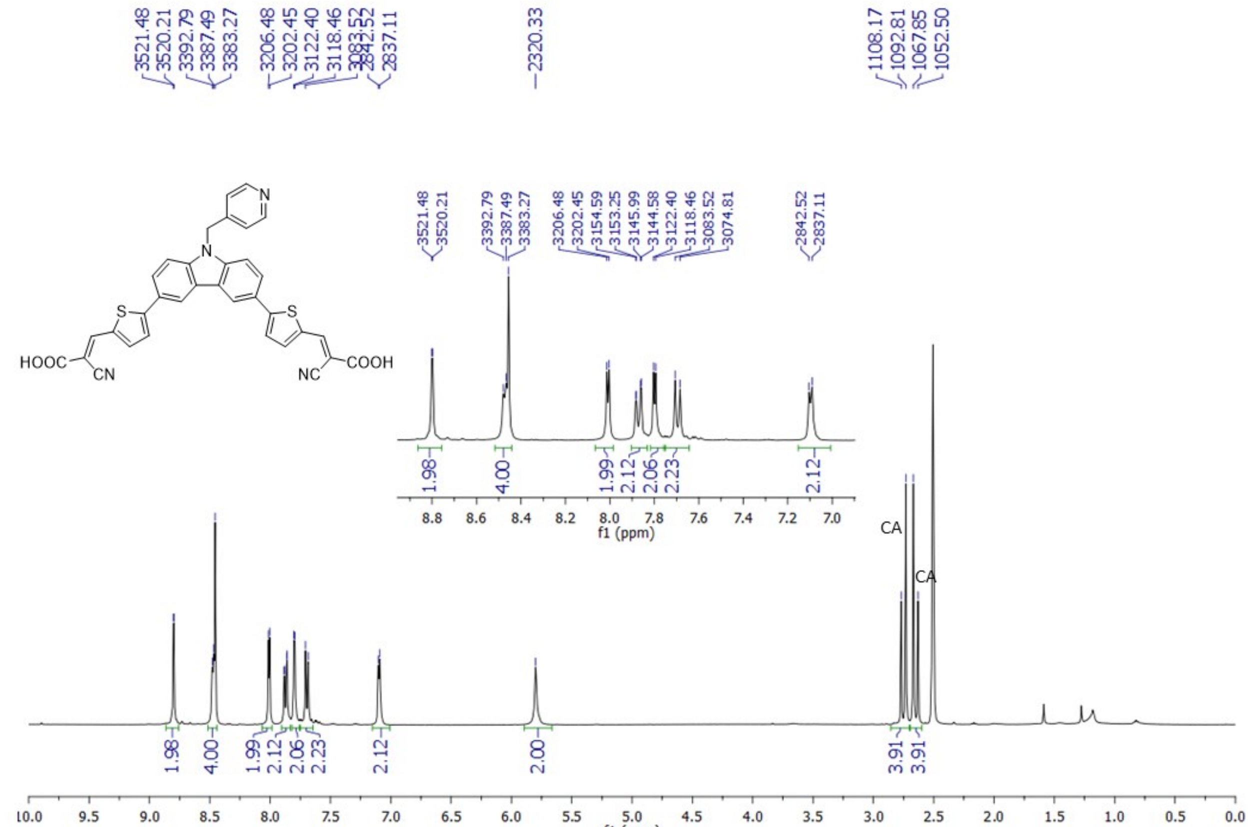

Figure S25. *^{1}H NMR of* ***CBZ-4Py*** *in DMSO-d$_6$.*

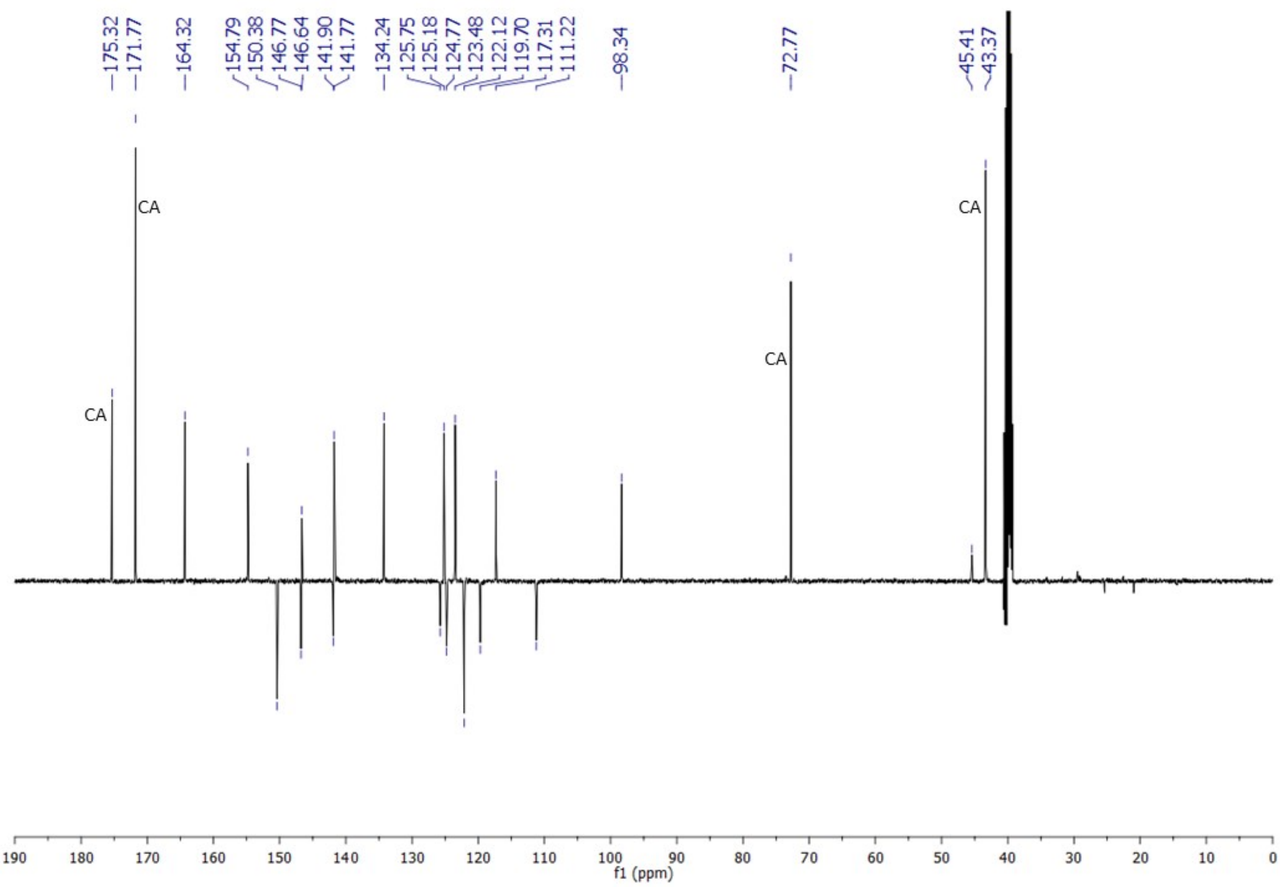

Figure S26. *^{13}C NMR of* ***CBZ-4Py*** *in DMSO-d$_6$.*

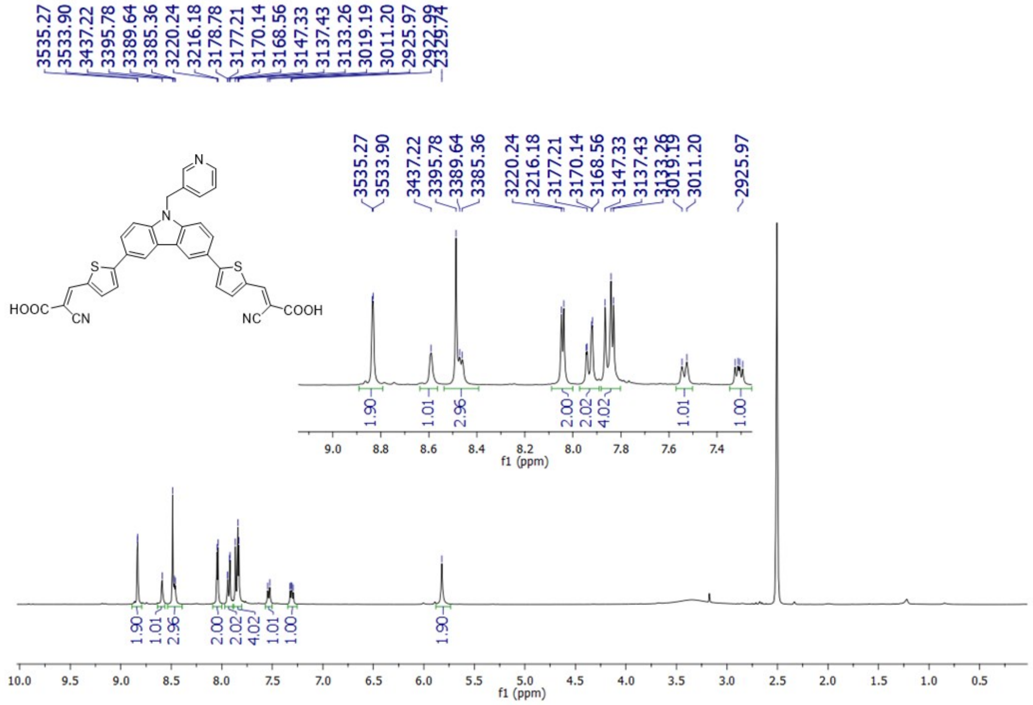

Figure S27. *^{1}H NMR of* ***CBZ-3Py*** *in DMSO-d$_6$.*

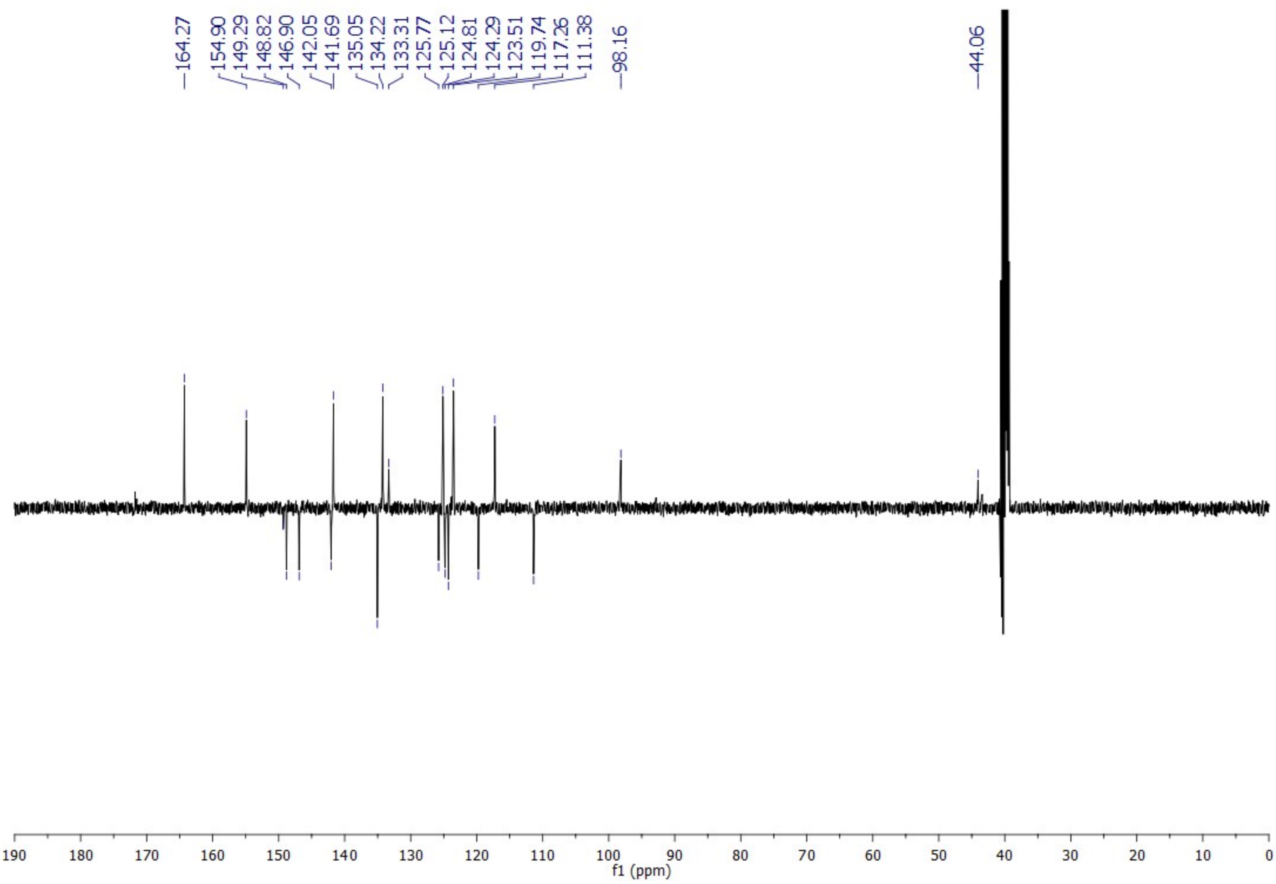

Figure S28. *^{13}C NMR of **CBZ-3Py** in DMSO-d$_6$.*

IR spectra

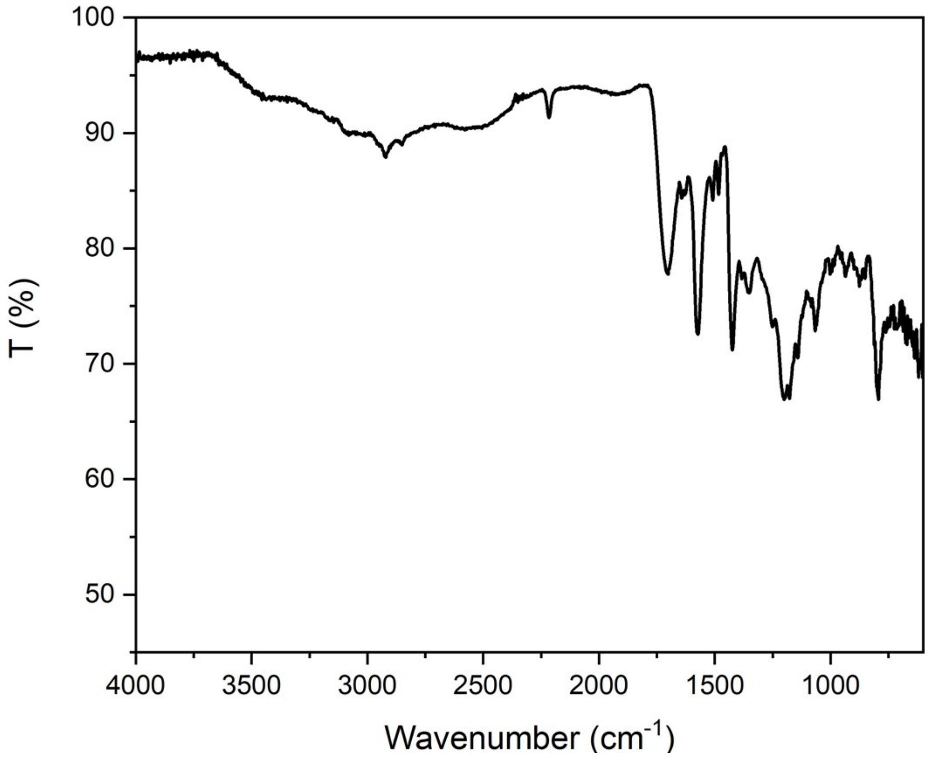

Figure S29**: FT-IR spectrum of **CBZ-4Py

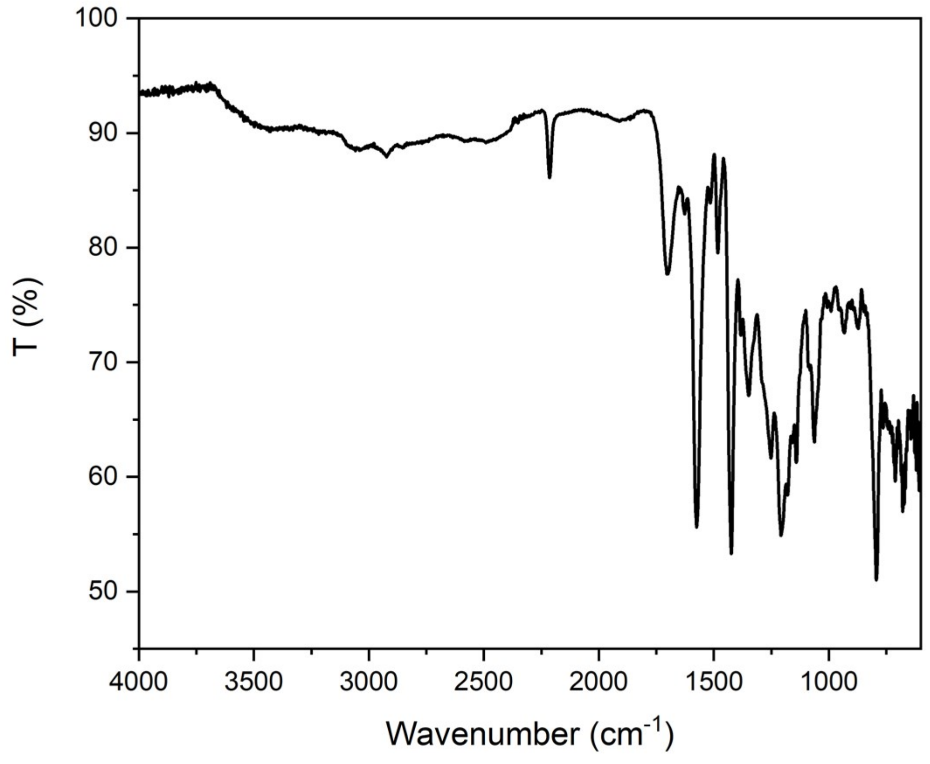

Figure S30**: FT-IR spectrum of **CBZ-3Py

HRMS spectra

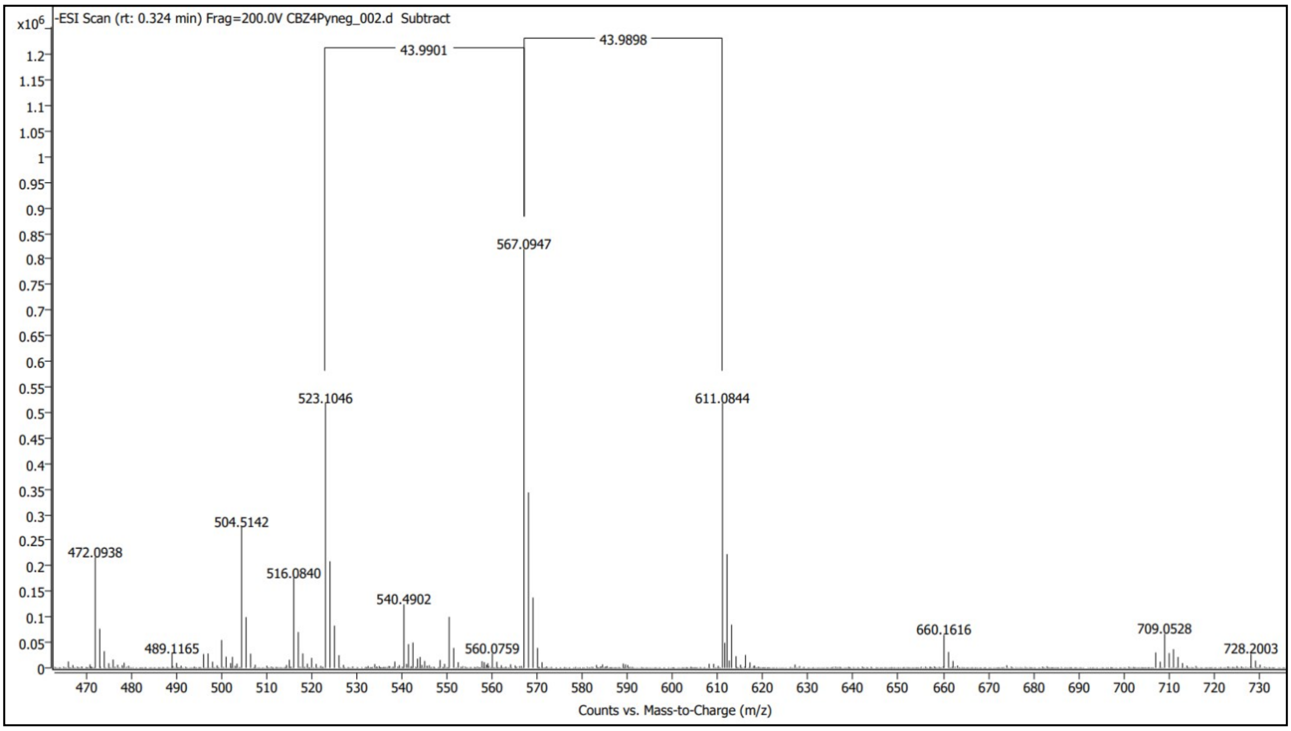

***Figure S31**. HRMS spectrum of **CBZ-4Py**.*

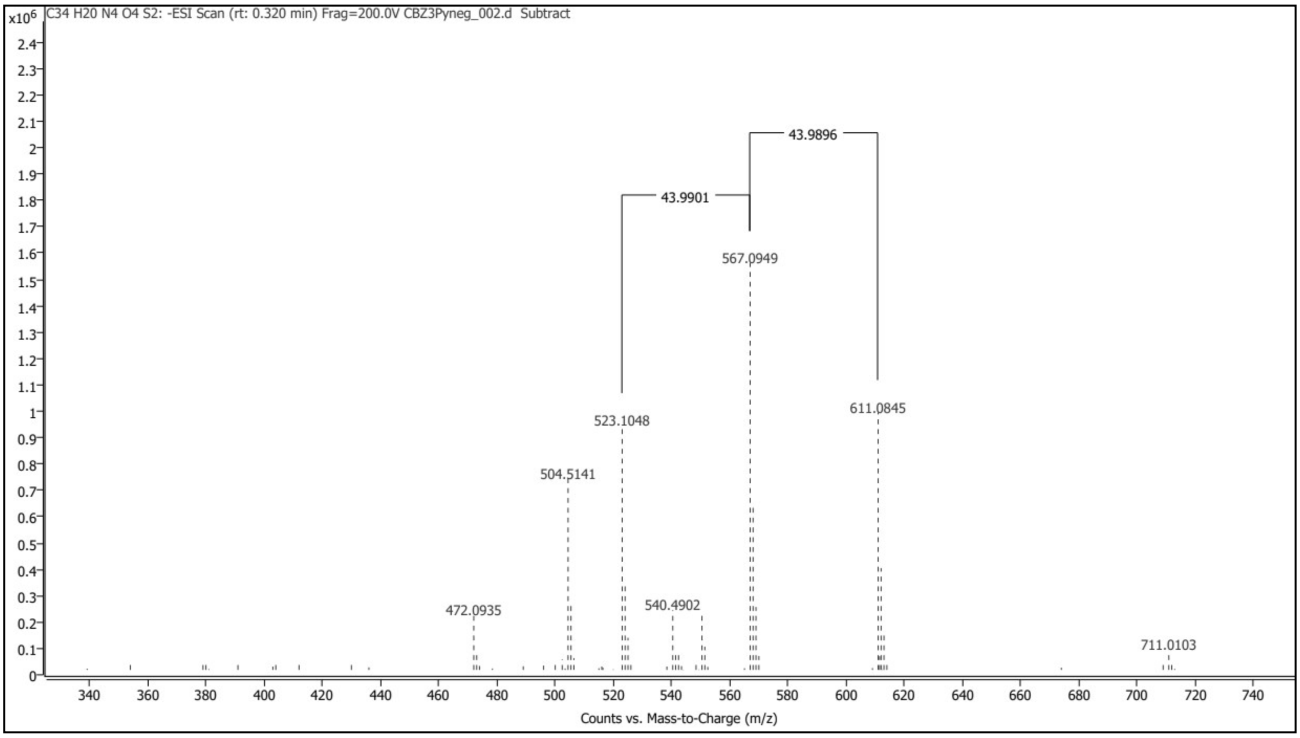

***Figure S32.** HRMS spectrum of **CBZ-3Py**.*
